# Global, regional, and national burden of neurological disorders, 1990–2016: a systematic analysis for the Global Burden of Disease Study 2016

**DOI:** 10.1016/S1474-4422(18)30499-X

**Published:** 2019-05

**Authors:** GBD 2016 Neurology Collaborators, Valery L Feigin, Emma Nichols, Tahiya Alam, Marlena S Bannick, Ettore Beghi, Natacha Blake, William J Culpepper, E Ray Dorsey, Alexis Elbaz, Richard G Ellenbogen, James L Fisher, Christina Fitzmaurice, Giorgia Giussani, Linda Glennie, Spencer L James, Catherine Owens Johnson, Nicholas J Kassebaum, Giancarlo Logroscino, Benoît Marin, W Cliff Mountjoy-Venning, Minh Nguyen, Richard Ofori-Asenso, Anoop P Patel, Marco Piccininni, Gregory A Roth, Timothy J Steiner, Lars Jacob Stovner, Cassandra E I Szoeke, Alice Theadom, Stein Emil Vollset, Mitchell Taylor Wallin, Claire Wright, Joseph Raymond Zunt, Nooshin Abbasi, Foad Abd-Allah, Ahmed Abdelalim, Ibrahim Abdollahpour, Victor Aboyans, Haftom Niguse Abraha, Dilaram Acharya, Abdu A Adamu, Oladimeji M Adebayo, Abiodun Moshood Adeoye, Jose C Adsuar, Mohsen Afarideh, Sutapa Agrawal, Alireza Ahmadi, Muktar Beshir Ahmed, Amani Nidhal Aichour, Ibtihel Aichour, Miloud Taki Eddine Aichour, Rufus Olusola Akinyemi, Nadia Akseer, Ayman Al-Eyadhy, Rustam Al-Shahi Salman, Fares Alahdab, Kefyalew Addis Alene, Syed Mohamed Aljunid, Khalid Altirkawi, Nelson Alvis-Guzman, Nahla Hamed Anber, Carl Abelardo T Antonio, Jalal Arabloo, Olatunde Aremu, Johan Ärnlöv, Hamid Asayesh, Rana Jawad Asghar, Hagos Tasew Atalay, Ashish Awasthi, Beatriz Paulina Ayala Quintanilla, Tambe B Ayuk, Alaa Badawi, Maciej Banach, Joseph Adel Mattar Banoub, Miguel A Barboza, Suzanne Lyn Barker-Collo, Till Winfried Bärnighausen, Bernhard T Baune, Neeraj Bedi, Masoud Behzadifar, Meysam Behzadifar, Yannick Béjot, Bayu Begashaw Bekele, Abate Bekele Belachew, Derrick A Bennett, Isabela M Bensenor, Adugnaw Berhane, Mircea Beuran, Krittika Bhattacharyya, Zulfiqar A Bhutta, Belete Biadgo, Ali Bijani, Nigus Bililign, Muhammad Shahdaat Bin Sayeed, Christopher Kynrint Blazes, Carol Brayne, Zahid A Butt, Ismael R Campos-Nonato, Carlos Cantu-Brito, Mate Car, Rosario Cárdenas, Juan J Carrero, Félix Carvalho, Carlos A Castañeda-Orjuela, Franz Castro, Ferrán Catalá-López, Ester Cerin, Yazan Chaiah, Jung-Chen Chang, Irini Chatziralli, Peggy Pei-Chia Chiang, Hanne Christensen, Devasahayam J Christopher, Cyrus Cooper, Paolo Angelo Cortesi, Vera M Costa, Michael H Criqui, Christopher Stephen Crowe, Albertino Antonio Moura Damasceno, Ahmad Daryani, Vanessa De la Cruz-Góngora, Fernando Pio De la Hoz, Diego De Leo, Gebre Teklemariam Demoz, Kebede Deribe, Samath Dhamminda Dharmaratne, Daniel Diaz, Mesfin Tadese Dinberu, Shirin Djalalinia, David Teye Doku, Manisha Dubey, Eleonora Dubljanin, Eyasu Ejeta Duken, David Edvardsson, Ziad El-Khatib, Matthias Endres, Aman Yesuf Endries, Sharareh Eskandarieh, Alireza Esteghamati, Sadaf Esteghamati, Farzaneh Farhadi, Andre Faro, Farshad Farzadfar, Mohammad Hosein Farzaei, Batool Fatima, Seyed-Mohammad Fereshtehnejad, Eduarda Fernandes, Garumma Tolu Feyissa, Irina Filip, Florian Fischer, Takeshi Fukumoto, Morsaleh Ganji, Fortune Gbetoho Gankpe, Miguel A Garcia-Gordillo, Abadi Kahsu Gebre, Teklu Gebrehiwo Gebremichael, Belayneh K Gelaw, Johanna M Geleijnse, Demeke Geremew, Kebede Embaye Gezae, Maryam Ghasemi-Kasman, Mahari Y Gidey, Paramjit Singh Gill, Tiffany K Gill, Efrata Tufa Girma, Elena V Gnedovskaya, Alessandra C Goulart, Ayman Grada, Giuseppe Grosso, Yuming Guo, Rahul Gupta, Rajeev Gupta, Juanita A Haagsma, Tekleberhan B Hagos, Arvin Haj-Mirzaian, Arya Haj-Mirzaian, Randah R Hamadeh, Samer Hamidi, Graeme J Hankey, Yuantao Hao, Josep Maria Haro, Hadi Hassankhani, Hamid Yimam Hassen, Rasmus Havmoeller, Simon I Hay, Mohamed I Hegazy, Behnam Heidari, Andualem Henok, Fatemeh Heydarpour, Chi Linh Hoang, Michael K Hole, Enayatollah Homaie Rad, Seyed Mostafa Hosseini, Guoqing Hu, Ehimario U Igumbor, Olayinka Stephen Ilesanmi, Seyed Sina Naghibi Irvani, Sheikh Mohammed Shariful Islam, Mihajlo Jakovljevic, Mehdi Javanbakht, Ravi Prakash Jha, Yash B Jobanputra, Jost B Jonas, Jacek Jerzy Jozwiak, Mikk Jürisson, Amaha Kahsay, Rizwan Kalani, Yogeshwar Kalkonde, Teshome Abegaz Kamil, Tanuj Kanchan, Manoochehr Karami, André Karch, Narges Karimi, Amir Kasaeian, Tesfaye Dessale Kassa, Zemenu Yohannes Kassa, Anil Kaul, Adane Teshome Kefale, Peter Njenga Keiyoro, Yousef Saleh Khader, Morteza Abdullatif Khafaie, Ibrahim A Khalil, Ejaz Ahmad Khan, Young-Ho Khang, Habibolah Khazaie, Aliasghar A Kiadaliri, Daniel N Kiirithio, Anthony S Kim, Daniel Kim, Young-Eun Kim, Yun Jin Kim, Adnan Kisa, Yoshihiro Kokubo, Ai Koyanagi, Rita V Krishnamurthi, Barthelemy Kuate Defo, Burcu Kucuk Bicer, Manasi Kumar, Ben Lacey, Alessandra Lafranconi, Van C Lansingh, Arman Latifi, Cheru Tesema Leshargie, Shanshan Li, Yu Liao, Shai Linn, Warren David Lo, Jaifred Christian F Lopez, Stefan Lorkowski, Paulo A Lotufo, Robyn M Lucas, Raimundas Lunevicius, Mark T Mackay, Narayan Bahadur Mahotra, Marek Majdan, Reza Majdzadeh, Azeem Majeed, Reza Malekzadeh, Deborah Carvalho Malta, Navid Manafi, Mohammad Ali Mansournia, Lorenzo Giovanni Mantovani, Winfried März, Tivani Phosa Mashamba-Thompson, Benjamin Ballard Massenburg, Kedar K V Mate, Colm McAlinden, John J McGrath, Varshil Mehta, Toni Meier, Hagazi Gebre Meles, Addisu Melese, Peter T N Memiah, Ziad A Memish, Walter Mendoza, Desalegn Tadese Mengistu, Getnet Mengistu, Atte Meretoja, Tuomo J Meretoja, Tomislav Mestrovic, Bartosz Miazgowski, Tomasz Miazgowski, Ted R Miller, GK Mini, Erkin M Mirrakhimov, Babak Moazen, Bahram Mohajer, Naser Mohammad Gholi Mezerji, Moslem Mohammadi, Maryam Mohammadi-Khanaposhtani, Roghayeh Mohammadibakhsh, Mousa Mohammadnia-Afrouzi, Shafiu Mohammed, Farnam Mohebi, Ali H Mokdad, Lorenzo Monasta, Stefania Mondello, Yoshan Moodley, Mahmood Moosazadeh, Ghobad Moradi, Maziar Moradi-Lakeh, Mehdi Moradinazar, Paula Moraga, Ilais Moreno Velásquez, Shane Douglas Morrison, Seyyed Meysam Mousavi, Oumer Sada Muhammed, Walter Muruet, Kamarul Imran Musa, Ghulam Mustafa, Mehdi Naderi, Gabriele Nagel, Aliya Naheed, Gurudatta Naik, Farid Najafi, Vinay Nangia, Ionut Negoi, Ruxandra Irina Negoi, Charles Richard James Newton, Josephine W Ngunjiri, Cuong Tat Nguyen, Long Hoang Nguyen, Dina Nur Anggraini Ningrum, Yirga Legesse Nirayo, Molly R Nixon, Bo Norrving, Jean Jacques Noubiap, Malihe Nourollahpour Shiadeh, Peter S Nyasulu, Okechukwu Samuel Ogah, In-Hwan Oh, Andrew T Olagunju, Tinuke O Olagunju, Pedro R Olivares, Obinna E Onwujekwe, Eyal Oren, Mayowa Ojo Owolabi, Mahesh PA, Amir H Pakpour, Wen-Harn Pan, Songhomitra Panda-Jonas, Jeyaraj Durai Pandian, Sangram Kishor Patel, David M Pereira, Max Petzold, Julian David Pillay, Michael A Piradov, Guilherme V Polanczyk, Suzanne Polinder, Maarten J Postma, Richie Poulton, Hossein Poustchi, Swayam Prakash, V Prakash, Mostafa Qorbani, Amir Radfar, Anwar Rafay, Alireza Rafiei, Fakher Rahim, Vafa Rahimi-Movaghar, Mahfuzar Rahman, Mohammad Hifz Ur Rahman, Muhammad Aziz Rahman, Fatemeh Rajati, Usha Ram, Anna Ranta, David Laith Rawaf, Salman Rawaf, Nickolas Reinig, Cesar Reis, Andre M N Renzaho, Serge Resnikoff, Shahab Rezaeian, Mohammad Sadegh Rezai, Carlos Miguel Rios González, Nicholas L S Roberts, Leonardo Roever, Luca Ronfani, Elias Merdassa Roro, Gholamreza Roshandel, Ali Rostami, Parisa Sabbagh, Ralph L Sacco, Perminder S Sachdev, Basema Saddik, Hosein Safari, Roya Safari-Faramani, Sare Safi, Saeid Safiri, Rajesh Sagar, Ramesh Sahathevan, Amirhossein Sahebkar, Mohammad Ali Sahraian, Payman Salamati, Saleh Salehi Zahabi, Yahya Salimi, Abdallah M Samy, Juan Sanabria, Itamar S Santos, Milena M Santric Milicevic, Nizal Sarrafzadegan, Benn Sartorius, Shahabeddin Sarvi, Brijesh Sathian, Maheswar Satpathy, Arundhati R Sawant, Monika Sawhney, Ione J C Schneider, Ben Schöttker, David C Schwebel, Soraya Seedat, Sadaf G Sepanlou, Hosein Shabaninejad, Azadeh Shafieesabet, Masood Ali Shaikh, Raad A Shakir, Mehran Shams-Beyranvand, Morteza Shamsizadeh, Mehdi Sharif, Mahdi Sharif-Alhoseini, Jun She, Aziz Sheikh, Kevin N Sheth, Mika Shigematsu, Rahman Shiri, Reza Shirkoohi, Ivy Shiue, Soraya Siabani, Tariq J Siddiqi, Inga Dora Sigfusdottir, Rannveig Sigurvinsdottir, Donald H Silberberg, João Pedro Silva, Dayane Gabriele Alves Silveira, Jasvinder A Singh, Dhirendra Narain Sinha, Eirini Skiadaresi, Mari Smith, Badr Hasan Sobaih, Soheila Sobhani, Moslem Soofi, Ireneous N Soyiri, Luciano A Sposato, Dan J Stein, Murray B Stein, Mark A Stokes, Mu'awiyyah Babale Sufiyan, Bryan L Sykes, PN Sylaja, Rafael Tabarés-Seisdedos, Braden James Te Ao, Arash Tehrani-Banihashemi, Mohamad-Hani Temsah, Omar Temsah, Jarnail Singh Thakur, Amanda G Thrift, Roman Topor-Madry, Miguel Tortajada-Girbés, Marcos Roberto Tovani-Palone, Bach Xuan Tran, Khanh Bao Tran, Thomas Clement Truelsen, Afewerki Gebremeskel Tsadik, Lorainne Tudor Car, Kingsley Nnanna Ukwaja, Irfan Ullah, Muhammad Shariq Usman, Olalekan A Uthman, Pascual R Valdez, Tommi Juhani Vasankari, Rajagopalan Vasanthan, Yousef Veisani, Narayanaswamy Venketasubramanian, Francesco S Violante, Vasily Vlassov, Kia Vosoughi, Giang Thu Vu, Isidora S Vujcic, Fasil Shiferaw Wagnew, Yasir Waheed, Yuan-Pang Wang, Elisabete Weiderpass, Jordan Weiss, Harvey A Whiteford, Tissa Wijeratne, Andrea Sylvia Winkler, Charles Shey Wiysonge, Charles D A Wolfe, Gelin Xu, Ali Yadollahpour, Tomohide Yamada, Yuichiro Yano, Mehdi Yaseri, Hiroshi Yatsuya, Ebrahim M Yimer, Paul Yip, Engida Yisma, Naohiro Yonemoto, Mahmoud Yousefifard, Chuanhua Yu, Zoubida Zaidi, Sojib Bin Zaman, Mohammad Zamani, Hamed Zandian, Zohreh Zare, Yunquan Zhang, Sanjay Zodpey, Mohsen Naghavi, Christopher J L Murray, Theo Vos

## Abstract

**Background:**

Neurological disorders are increasingly recognised as major causes of death and disability worldwide. The aim of this analysis from the Global Burden of Diseases, Injuries, and Risk Factors Study (GBD) 2016 is to provide the most comprehensive and up-to-date estimates of the global, regional, and national burden from neurological disorders.

**Methods:**

We estimated prevalence, incidence, deaths, and disability-adjusted life-years (DALYs; the sum of years of life lost [YLLs] and years lived with disability [YLDs]) by age and sex for 15 neurological disorder categories (tetanus, meningitis, encephalitis, stroke, brain and other CNS cancers, traumatic brain injury, spinal cord injury, Alzheimer's disease and other dementias, Parkinson's disease, multiple sclerosis, motor neuron diseases, idiopathic epilepsy, migraine, tension-type headache, and a residual category for other less common neurological disorders) in 195 countries from 1990 to 2016. DisMod-MR 2.1, a Bayesian meta-regression tool, was the main method of estimation of prevalence and incidence, and the Cause of Death Ensemble model (CODEm) was used for mortality estimation. We quantified the contribution of 84 risks and combinations of risk to the disease estimates for the 15 neurological disorder categories using the GBD comparative risk assessment approach.

**Findings:**

Globally, in 2016, neurological disorders were the leading cause of DALYs (276 million [95% UI 247–308]) and second leading cause of deaths (9·0 million [8·8–9·4]). The absolute number of deaths and DALYs from all neurological disorders combined increased (deaths by 39% [34–44] and DALYs by 15% [9–21]) whereas their age-standardised rates decreased (deaths by 28% [26–30] and DALYs by 27% [24–31]) between 1990 and 2016. The only neurological disorders that had a decrease in rates and absolute numbers of deaths and DALYs were tetanus, meningitis, and encephalitis. The four largest contributors of neurological DALYs were stroke (42·2% [38·6–46·1]), migraine (16·3% [11·7–20·8]), Alzheimer's and other dementias (10·4% [9·0–12·1]), and meningitis (7·9% [6·6–10·4]). For the combined neurological disorders, age-standardised DALY rates were significantly higher in males than in females (male-to-female ratio 1·12 [1·05–1·20]), but migraine, multiple sclerosis, and tension-type headache were more common and caused more burden in females, with male-to-female ratios of less than 0·7. The 84 risks quantified in GBD explain less than 10% of neurological disorder DALY burdens, except stroke, for which 88·8% (86·5–90·9) of DALYs are attributable to risk factors, and to a lesser extent Alzheimer's disease and other dementias (22·3% [11·8–35·1] of DALYs are risk attributable) and idiopathic epilepsy (14·1% [10·8–17·5] of DALYs are risk attributable).

**Interpretation:**

Globally, the burden of neurological disorders, as measured by the absolute number of DALYs, continues to increase. As populations are growing and ageing, and the prevalence of major disabling neurological disorders steeply increases with age, governments will face increasing demand for treatment, rehabilitation, and support services for neurological disorders. The scarcity of established modifiable risks for most of the neurological burden demonstrates that new knowledge is required to develop effective prevention and treatment strategies.

**Funding:**

Bill & Melinda Gates Foundation.

## Introduction

The UN General Assembly report of December, 2017, underscored that progress in reducing the burden of non-communicable diseases, including neurological disorders, has been insufficient to meet the UN Sustainable Development Goal targets by 2030.^1^ Neurological disorders are the leading cause of disability and the second leading cause of death worldwide.^2^ Thus, strategies and programmes that reduce the burden from neurological disorders would potentially help to achieve the UN Sustainable Development Goal targets.

Although age-standardised incidence, mortality, and prevalence rates of many neurological disorders declined for many countries from 1990 to 2015, the absolute number of people affected by, dying, or remaining disabled from neurological disorders over the past 25 years has been increasing globally.^2^ Regular assessments of incidence, prevalence, mortality, and disability associated with neurological disorders by cause and over time are important for evidence-based health-care planning, priority setting, and resource allocation. On a global scale, this type of information has facilitated between-country and between-region comparisons of the burden from neurological disorders and their trends. The information can also be used for hypothesis generation on causal relationships and effects of sociodemographic and health care factors on the burden from neurological disorders.

**Research in context**

**Evidence before this study**

Findings from the Global Burden of Diseases, Injuries, and Risk Factors Study (GBD) 2015, showed that neurological disorders are the world's largest cause of disability. However, some disabling neurological disorders, specifically traumatic brain injury and spinal cord injury, were not included, thus underestimating the global burden of this group of disabilities. No other relevant efforts, beyond GBD, have been made to quantify the global burden of neurological disorders as a whole. The data sources identified after the GBD 2015 study are detailed in the other GBD papers in the series of the neurological disorders overvieved in this paper.

**Added value of this study**

This study improves upon GBD 2015 by including four significant considerations. First, compared with a previous analysis based on GBD 2015, we were able to add the non-fatal outcomes of traumatic brain injury and spinal cord injury. Thus, in this analysis, we have aggregated results from GBD 2016 for 15 disease and injury outcomes that are generally cared for by neurological services, such as CNS infectious conditions (tetanus, meningitis, and encephalitis), stroke, brain and other CNS cancers, traumatic brain injury and spinal cord injury, Alzheimer's disease and other dementias, Parkinson's disease, multiple sclerosis, motor neuron diseases, idiopathic epilepsy, migraine, tension-type headache, and a residual category of other less common neurological disorders. Second, we incorporated new data from sources identified after GBD 2015, including peer-reviewed publications, reports from statistical agencies or ministries of health, surveys, and administrative and hospital data. Third, in the individual papers of the burden of neurological disorders analysed, we extended our analysis of GBD results by Socio-demographic Index (SDI), a summary indicator of income per capita, years of schooling, and total fertility rate, with new ways of presenting and visualising changes over time and the relationship with SDI. Fourth, we extended the terminal age group used in our analysis from 80 years and older to 80–84 years, 85–89 year, 90–94 year, and 95 years and older.

**Implications of all the available evidence**

Neurological disorders continue to be the leading cause of disability worldwide, and their contribution to the overall burden from all health conditions is increasing. The bulk of the burden from neurological disorders continues to be in low-income and middle-income countries. Ageing of the population, population growth, and ongoing epidemiological transition across all countries require regular updates on the global, regional, and national burden from neurological disorders to allow evidence-based health-care planning and resource allocation for these disorders.

In 2017, we published aggregated estimates of the burden from neurological disorders^2^ (as measured by prevalence, mortality, disability-adjusted life-years [DALYs], and years lived with disability [YLDs]), based on the Global Burden of Diseases, Injuries, and Risk Factors Study (GBD) 2015 data. However, neurological disorders analysed did not include traumatic brain injury (TBI) or spinal cord injury, which are significant sources of global disability. Additionally, newly available publications and other data sources were added to the GBD 2016 estimates.

In the 2018–19 series of publications in *The Lancet Neurology*,^3^, ^4^, ^5^, ^6^, ^7^, ^8^, ^9^, ^10^, ^11^, ^12^ we presented global, regional, and national estimates of the burden of individual neurological disorders as measured by prevalence, mortality, DALYs, YLDs, years of life lost (YLLs), and, for selected disorders, also incidence, and their trends from 1990 to 2016 according to Socio-demographic Index (SDI), a summary indicator of income per capita, years of schooling, and total fertility rate. Here, we present updated aggregated estimates (compared with aggregated estimates presented in the GBD 2015 study) of neurological disorders and their trends from 1990 to 2016 by SDI, age, and sex.

## Methods

### Overview

The Institute for Health Metrics and Evaluation produces annual updates of the GBD study and includes a growing collaboration of scientists. Reported estimates span the period from 1990 to 2016. Annual updates allow incorporation of new data and methodological improvements to ensure that the most up-to-date information is available to policy makers to help make resource allocation decisions. In this analysis, we have aggregated results from GBD 2016 for 15 disease and injury outcomes that are generally cared for by neurological services. These include infectious conditions (tetanus, meningitis, and encephalitis), stroke, brain and other CNS cancers, TBI, spinal cord injury, Alzheimer's disease and other dementias, Parkinson's disease, multiple sclerosis, motor neuron diseases, idiopathic epilepsy, migraine, tension-type headache, and a residual category of other less common neurological disorders. Compared to a previous analysis based on GBD 2015,^2^ we added non-fatal outcomes of TBI and spinal cord injury. Medication overuse headache is no longer included as a separate cause but quantified as a consequence of the underlying headache types. For all neurological disorders combined we report here estimates of deaths and DALYs because aggregate incidence and prevalence estimates of combined neurological disorders are not useful for policy making.

In the methods section of this overview paper, we present a summary of the general methods of the GBD as they apply to neurological disorders. In the ten accompanying disease-specific papers, we have concentrated on methods that are specific to each disorder. Details on the methods of estimates for tetanus, encephalitis, and the residual category of other neurological disorders are provided in the appendix. The guiding principle of GBD is to assess health loss due to mortality and disability comprehensively, defining disability as any departure from full health. In GBD 2016, estimates were made for 195 countries and territories and 579 subnational locations, for 27 years starting from 1990, for 23 age groups, and for both sexes. Deaths were estimated for 264 diseases and injuries, whereas prevalence and incidence were estimated for 328 diseases and injuries. To allow meaningful comparisons between deaths and non-fatal disease outcomes as well as between diseases, data for deaths from and prevalence of neurological disorders are summarised in a single indicator, the DALY. DALYs are the sum of YLLs and YLDs. YLLs are estimated as the product of counts of deaths and a standard ideal remaining life expectancy at the age of death. The standard life expectancy is derived from the lowest observed mortality rates by age in any population in the world larger than 5 million people.^13^ YLDs are estimated as the product of prevalence of individual consequences of disease (or sequelae) multiplied by their corresponding disability weights, which quantify the relative severity of sequelae as a number between 0 (representing full health) and 1 (representing death). Disability weights have been estimated in nine population surveys and an open-access internet survey in which respondents were asked to choose the healthier option between random pairs of health states that were presented with short descriptions of their main features.^14^

### Mortality estimates

All-cause mortality rates are estimated from vital registration data in countries with complete coverage as analysed by demographic growth balance methods comparing successive census population counts.^15^ For other countries, the probabilities of death before age 5 years and between ages 15 and 60 years are estimated from censuses and surveys asking mothers to provide a history of children ever born and those still alive, and surveys asking adults about siblings who are alive or have died. Using model life tables, these probabilities of death are transformed into age-specific death rates by location, year, and sex. GBD has collated a large database of cause-of-death data from vital registrations and verbal autopsy surveys in which relatives are asked a standard set of questions to ascertain the likely cause of death, supplemented with police and mortuary data for injury deaths in countries with no other data. Cause-of-death information is provided in a large number of different classification systems based on versions of the International Classification of Diseases (ICD) or bespoke classifications in some countries. All data are mapped into the disease and injury categories of GBD. All classification systems contain codes that are less informative than ICD because they do not have a specific diagnosis (eg, unspecified cancer) or refer to codes that cannot be the underlying cause of death (eg, low back pain or senility) or are intermediate causes (eg, heart failure or sepsis). Such deaths are redistributed to more precise underlying causes of death.^13^ After these redistributions and corrections for under-registration, the data are analysed with the Cause of Death Ensemble model (CODEm), a highly systematised tool that runs many different models on the same data and chooses an ensemble of models that best reflects all the available input data. The statistical performance of all models is tested by withholding 30% of the data and checking how well a model covers the data that were held out. To enforce consistency from CODEm, the sum of all-cause specific mortality rates is scaled to that of the all-cause mortality rates in each age, sex, location, and year category. All our estimates of causes of death are categorical: each death is assigned to a single underlying cause. This means that all estimates add to 100%.

### Non-fatal estimates

Non-fatal estimates are based on systematic reviews of published papers and unpublished documents, survey microdata, administrative records of health encounters, registries, and disease surveillance systems. These data sources are catalogued in our Global Health Data Exchange (GHDx), the largest repository of health data globally. We first set a reference case definition or study method, or both, that best quantifies each disease or injury or consequence thereof. If there is evidence of a systematic bias in data that used different case definitions or methods compared to reference data, we adjust those datapoints to reflect what their value would have been if measured as the reference. DisMod-MR 2.1, a Bayesian meta-regression tool, is our main method of analysing non-fatal data. It is designed as a geographical cascade where a first model is run on data from all countries, which produces an initial global fit and estimates coefficients for predictor variables and the adjustments for alternative study characteristics. The global fit adjusted by the values of random effects for each of seven GBD super-regions (appendix), the coefficients on sex, and country predictors, is passed down as data to a model for each super-region together with the input data for that geography. The same steps are repeated going from super-regions to 21 regions and then to 195 countries and, where applicable, a further level down to subnational units. Below the global level, all models are run separately by sex and for six time periods (1990, 1995, 2000, 2005, 2010, and 2016). During each analysis, all data for prevalence, incidence, remission (ie, cure rate), and mortality are forced to be internally consistent. For most diseases, the bulk of data for prevalence or incidence are at the disease level, with even fewer studies providing data on the proportions of cases of disease in each of the sequelae defined for the disease. The proportions in each sequela are pooled using DisMod-MR 2.1 or meta-analysis, or are derived from analyses of patient-level datasets. The multiplication of prevalent cases for each disease sequela and the appropriate disability weight produces YLD estimates that do not yet take into account comorbidity. To correct for comorbidity, these data are used in a simulation to create hypothetical individuals in each age, sex, location, and year combination who have none, one, or multiple sequelae simultaneously. We assume that disability weights are multiplicative rather than additive because this avoids assigning a combined disability weight value in any individual exceeding 1—ie, worse off than a year lost due to death.

### Risk factors

For risks, we use a different, counterfactual approach—ie, answering the question “what would the burden have been if the population had been exposed to a theoretical minimum level of exposure to a risk?” Thus, we need to define what level of exposure to a risk factor leads to the lowest amount of disease. We then analyse data on the prevalence of exposure to a risk and derive relative risks for any risk–outcome pair for which we find sufficient evidence of a causal relationship.^16^ Prevalence of exposure is estimated in DisMod-MR 2.1 using spatiotemporal Gaussian process regression, or from satellite imagery in the case of ambient air pollution. Relative risk data are pooled using meta-analysis of cohort, case-control, and intervention studies. From the prevalence and relative risk results, population attributable fractions are estimated relative to the theoretical minimum risk exposure level (TMREL). Criteria for inclusion of risks into GBD were the availability of sufficient evidence for a causal relationship between a risk and one or more disease or injury outcomes; evidence to support generalisability of an effect size beyond the populations included in epidemiological studies; availability of sufficient data and methods to enable estimation of exposure levels by country; and the likely importance of a risk factor to disease burden or policy considerations.^17^

### Uncertainty intervals (UIs)

Uncertainty is propagated throughout all these calculations by creating 1000 values for each prevalence, death, YLL, YLD, or DALY estimate and performing aggregations across causes and locations at the level of each of the 1000 values for all intermediate steps in the calculation. The lower and upper bounds of the 95% UI are the 25th and 975th values of the ordered 1000 values. Significance of differences was established if 975 or more of the ordered 1000 values of difference were on either side of zero.

### SDI

GBD uses a composite indicator of sociodemographic development, SDI, which reflects the geometric mean of normalised values of a location's income per capita, the average years of schooling in the population aged 15 years and older, and the total fertility rate. Countries and territories are grouped into quintiles of high, high-middle, middle, low-middle, and low SDI based on their 2016 values.^13^

### Role of the funding source

The funder of the study had no role in study design, data collection, data analysis, data interpretation, or the writing of the report. All authors had full access to the data in the study and had final responsibility for the decision to submit for publication.

## Results

In 2016, the neurological disorders included in this analysis were responsible for 276 million (95% UI 247–308) DALYs, comprising 11·6% (10·7–12·4) of global DALYs for all diseases. Combined, these disorders were the underlying cause in 9·0 million (8·8–9·4) deaths or 16·5% (16·1–17·0) of total global deaths in 2016. Incidence and prevalence estimates for each neurological disorder can be found in the GBD online results tool. Results and findings mentioned can also be viewed interactively through an online data visualisation tool. Neurological disorders were the leading cause group of global DALYs in 2016, followed by cardiovascular diseases (excluding stroke). In terms of deaths, these combined neurological disorders ranked second after cardiovascular diseases. Since 1990, the number of deaths from neurological disorders increased by 39% (34–44) and DALYs for neurological disorders increased by 15% (9–21). However, age-standardised mortality rates decreased by 28% (26–30) over the same time, indicating that population increase and ageing are driving global numbers higher, even though the global population is exposed to a lower risk of death from these causes, as indicated by the lower age-standardised rates. Similarly, the age-standardised DALY rates decreased by 27% (24–31) between 1990 and 2016. The only neurological disorders that had a decrease in rates and absolute numbers of deaths and DALYs were tetanus, meningitis, and encephalitis.

**Figure 1:**
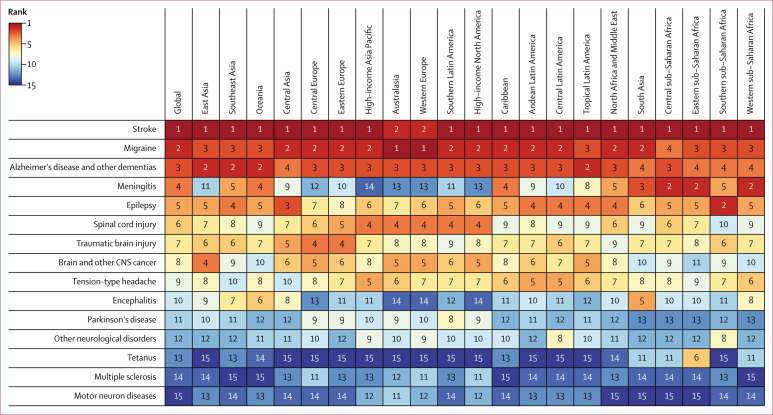
Ranking of age-standardised DALY rates for all neurological disorders by region, 2016 DALY=disability-adjusted life-year.

Stroke was the largest contributor to global neurological DALYs, responsible for 42·2% (95% UI 38·6–46·1) of these DALYs in 2016 (table 1). Migraine was the second largest contributor (16·3% [11·7–20·8]), followed by Alzheimer's and other dementias (10·4% [9·0–12·1]) and meningitis (7·9% [6·6–10·4]). Stroke ranked first among neurological disorders in terms of age-standardised DALY rates in 19 of 21 world regions (figure 1). In Australasia and western Europe, migraine ranked first. Migraine and Alzheimer's disease and other dementias were ranked among the top four contributing neurological conditions in all 21 GBD world regions. Large variations in ranking were found for meningitis (ranked second in central, eastern, and western sub-Saharan Africa and 14th in high-income Asia Pacific), epilepsy (ranked second in southern sub-Saharan Africa and third in central Asia and eighth in eastern Europe), and encephalitis (ranked fifth in south Asia and 14th in Australasia, western Europe, and high-income North America). Other high rankings of note are for spinal cord injury (ranked fourth in all five high-income regions), TBI (ranked fourth in central and eastern Europe), and tetanus (ranked sixth in eastern sub-Saharan Africa; figure 1).

**Table 1:** Global deaths, DALYs, incidence, and prevalence per 100 000 people and age-standardised rates by neurological disorder category, 1990–2016

	**Absolute numbers (thousands)**		**Age-standardised rate (per 100 000 people)**				
	2016	Percentage change, 1990–2016	2016	Percentage change, 1990–2016	Males	Females	Male-to-female ratio
**All neurological disorders**							
Deaths	9039 (8772 to 9364)	39% (34 to 44)	144 (140 to 149)	−28% (−30 to −26)	160 (155 to 166)	129 (124 to 135)	1·24 (1·20 to 1·29)
DALYs	276 143 (246 544 to 307 994)	15% (9 to 21)	3968 (3557 to 4396)	−27% (−31 to −24)	4204 (3855 to 4575)	3755 (3272 to 4279)	1·12 (1·05 to 1·20)
**Tetanus**							
Deaths	37 (22 to 47)	−89% (−91 to −86)	1 (0 to 1)	−91% (−93 to −88)	1 (0 to 1)	0 (0 to 1)	1·55 (0·85 to 2·15)
DALYs	2367 (1446 to 3063)	−90% (−93 to −88)	34 (20 to 43)	−91% (−93 to −89)	41 (21 to 56)	26 (16 to 35)	1·59 (0·93 to 2·25)
Incidence	90 (51 to 121)	−89% (−92 to −86)	1 (1 to 2)	−91% (−93 to −88)	2 (1 to 2)	1 (1 to 1)	1·72 (0·98 to 2·37)
**Meningitis**							
Deaths	318 (265 to 409)	−21% (−36 to 9)	5 (4 to 6)	−36% (−47 to −12)	5 (4 to 7)	4 (3 to 6)	1·25 (0·86 to 1·86)
DALYs	21 866 (18 205 to 28 281)	−28% (−42 to 3)	306 (254 to 398)	−36% (−48 to −10)	328 (261 to 428)	284 (224 to 423)	1·18 (0·79 to 1·66)
Incidence	2821 (2464 to 3310)	13% (10 to 16)	39 (35 to 46)	−4% (−7 to −1)	42 (36 to 49)	37 (32 to 44)	1·12 (1·10 to 1·14)
**Encephalitis**							
Deaths	103 (84 to 138)	−2% (−36 to 70)	1 (1 to 2)	−27% (−51 to 21)	2 (1 to 2)	1 (1 to 2)	1·19 (0·81 to 1·57)
DALYs	6704 (5469 to 8574)	−15% (−44 to 41)	93 (76 to 118)	−32% (−54 to 10)	92 (75 to 124)	93 (74 to 124)	0·99 (0·73 to 1·26)
Incidence	6534 (5957 to 7165)	29% (26 to 33)	90 (82 to 98)	−5% (−6 to −4)	82 (75 to 90)	99 (90 to 109)	0·83 (0·80 to 0·85)
**Stroke**							
Deaths	5528 (5335 to 5735)	28% (22 to 33)	87 (83 to 90)	−36% (−39 to −34)	103 (99 to 107)	72 (69 to 77)	1·42 (1·35 to 1·50)
DALYs	116 445 (111 385 to 121 407)	22% (17 to 27)	1711 (1635 to 1784)	−34% (−37 to −32)	2046 (1961 to 2126)	1408 (1320 to 1489)	1·45 (1·39 to 1·52)
Incidence	13 677 (12 713 to 14 692)	78% (73 to 83)	203 (189 to 218)	−8% (−11 to −6)	231 (215 to 248)	179 (166 to 192)	1·29 (1·27 to 1·31)
**Alzheimer's disease and other dementias**							
Deaths	2382 (2060 to 2778)	148% (140 to 157)	41 (35 to 48)	4% (1 to 6)	37 (32 to 44)	43 (37 to 49)	0·88 (0·86 to 0·91)
DALYs	28 764 (24 511 to 33 952)	121% (115 to 127)	471 (401 to 556)	2% (0 to 4)	439 (373 to 523)	490 (417 to 576)	0·90 (0·88 to 0·92)
Prevalence	43 836 (37 756 to 51 028)	117% (114 to 121)	712 (614 to 828)	2% (1 to 2)	645 (555 to 752)	757 (652 to 879)	0·85 (0·85 to 0·86)
**Parkinson's disease**							
Deaths	211 (168 to 265)	161% (152 to 171)	3 (3 to 4)	19% (16 to 23)	5 (4 to 6)	3 (2 to 3)	1·81 (1·74 to 1·89)
DALYs	3235 (2564 to 4013)	148% (140 to 156)	51 (41 to 63)	22% (18 to 26)	67 (53 to 83)	39 (31 to 49)	1·70 (1·63 to 1·76)
Prevalence	6063 (4971 to 7325)	145% (138 to 152)	94 (77 to 114)	22% (18 to 25)	112 (92 to 135)	80 (65 to 97)	1·40 (1·36 to 1·43)
**Idiopathic epilepsy**							
Deaths	126 (119 to 136)	9% (−3 to 30)	2 (2 to 2)	−24% (−32 to −11)	2 (2 to 2)	1 (1 to 2)	1·50 (1·37 to 1·68)
DALYs	13 492 (11 015 to 16 503)	9% (−3 to 24)	183 (149 to 223)	−19% (−28 to −9)	201 (167 to 241)	164 (131 to 204)	1·23 (1·16 to 1·31)
Prevalence	23 962 (20 402 to 27 737)	48% (34 to 63)	327 (278 to 378)	6% (−4 to 17)	334 (286 to 387)	320 (272 to 372)	1·04 (1·02 to 1·06)
**Multiple sclerosis**							
Deaths	19 (17 to 21)	61% (19 to 74)	0 (0 to 0)	−12% (−35 to −5)	0 (0 to 0)	0 (0 to 0)	0·74 (0·63 to 0·91)
DALYs	1151 (969 to 1346)	66% (45 to 75)	16 (13 to 18)	−4% (−16 to 1)	12 (10 to 14)	19 (16 to 23)	0·60 (0·54 to 0·67)
Prevalence	2221 (2034 to 2437)	88% (85 to 90)	30 (28 to 33)	10% (9 to 12)	19 (18 to 21)	41 (37 to 45)	0·48 (0·46 to 0·49)
**Migraine**							
DALYs	45 122 (29 046 to 62 827)	51% (50 to 53)	599 (386 to 833)	0% (−1 to 0)	422 (274 to 587)	778 (500 to 1084)	0·54 (0·53 to 0·56)
Prevalence	1044 771 (999 535 to 1087 969)	48% (47 to 49)	13 847 (13 255 to 14 418)	−2% (−2 to −2)	9356 (8962 to 9753)	18 408 (17 623 to 19 194)	0·51 (0·50 to 0·51)
**Tension-type headache**							
DALYs	7195 (4615 to 10 500)	53% (47 to 58)	96 (62 to 140)	0% (−3 to 2)	77 (50 to 113)	115 (74 to 167)	0·68 (0·67 to 0·68)
Prevalence	1890 670 (1707 786 to 2097 762)	37% (35 to 39)	25 130 (22 741 to 27 895)	−7% (−8 to −7)	20 369 (18 312 to 22 765)	29 962 (27 231 to 33 041)	0·68 (0·67 to 0·69)
**Motor neuron diseases**							
Deaths	34 (33 to 35)	94% (80 to 102)	1 (0 to 1)	8% (0 to 12)	1 (1 to 1)	0 (0 to 0)	1·46 (1·36 to 1·54)
DALYs	926 (882 to 962)	59% (45 to 72)	13 (13 to 14)	−2% (−9 to 3)	16 (15 to 16)	11 (10 to 11)	1·47 (1·36 to 1·54)
Prevalence	331 (300 to 367)	67 % (62 to 72)	5 (4 to 5)	5% (3 to 6)	5 (5 to 6)	4 (4 to 5)	1·25 (1·23 to 1·28)
**Brain and other CNS cancer**							
Deaths	227 (205 to 241)	64% (54 to 82)	3 (3 to 3)	−2% (−8 to 8)	4 (3 to 4)	3 (2 to 3)	1·41 (1·21 to 1·65)
DALYs	7660 (6923 to 8280)	36% (26 to 55)	105 (95 to 113)	−10% (−16 to 3)	122 (106 to 135)	88 (77 to 97)	1·39 (1·18 to 1·64)
Prevalence	781 (693 to 818)	97% (84 to 115)	11 (10 to 12)	25% (17 to 34)	12 (10 to 13)	10 (9 to 11)	1·14 (0·98 to 1·31)
**Traumatic brain injuries**							
DALYs	8000 (5856 to 10 108)	77% (17 to 157)	111 (82 to 141)	9% (8 to 9)	141 (104 to 180)	82 (61 to 101)	1·76 (1·16 to 2·58)
Incidence	27 082 (24 302 to 30 299)	47% (44 to 51)	369 (331 to 412)	4% (2 to 5)	471 (427 to 520)	264 (232 to 301)	1·79 (1·70 to 1·88)
**Spinal cord injuries**							
DALYs	9522 (6700 to 12 449)	40% (36 to 44)	130 (90 to 170)	−10% (−13 to −7)	145 (100 to 188)	113 (80 to 146)	1·29 (1·11 to 1·48)
Incidence	935 (781 to 1155)	39% (33 to 49)	13 (11 to 16)	−4% (−7 to 4)	15 (12 to 18)	11 (9 to 14)	1·37 (1·27 to 1·47)
**Other neurological disorders**							
Deaths	53 (51 to 55)	66% (51 to 78)	1 (1 to 1)	1% (−5 to 5)	1 (1 to 1)	1 (1 to 1)	1·42 (1·29 to 1·54)
DALYs	3695 (3114 to 4353)	54% (39 to 68)	51 (43 to 60)	11% (2 to 20)	55 (47 to 64)	46 (38 to 55)	1·21 (1·13 to 1·31)

Numbers in brackets are 95% uncertainty intervals. DALYs for traumatic brain injuries and spinal cord injuries include years lived with disability only (not years of life lost), because International Classification of Disease rules for cause of death reporting require that injury deaths are assigned to causes rather than consequences. We report incidence or prevalence depending on what is the most commonly used or relevant measure of frequency. Prevalence estimates of other neurological disorders and all neurological disorders were not computed because aggregates of prevalence among disparate disease entities are not very informative to policy. DALYS=disability-adjusted life-years.

**Figure 2:**
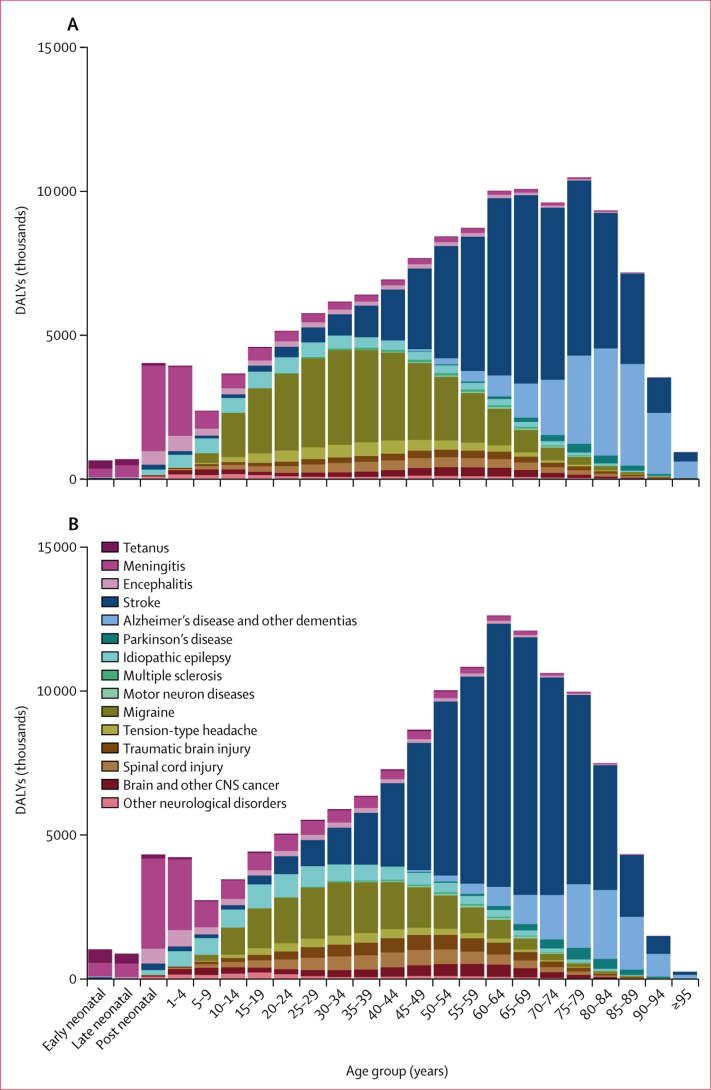
Global DALYs for neurological disorders by sex and age, 2016 Early neonatal is 0–7 days; late neonatal is 7–28 days; and post-neonatal is 28 days to 1 year. (A) Females. (B) Males. DALY=disability-adjusted life-year.

Across the aggregate of 15 neurological disorder categories analysed, age-standardised DALY rates were significantly higher in males than in females (male-to-female ratio 1·12 [95% UI 1·05–1·20]). When examining individual disorders, a significantly higher burden (as measured by age-standardised DALY rates) in males than females was observed for TBI, Parkinson's disease, tetanus, motor neuron diseases, and stroke, with male-to-female ratios of at least 1·5. By contrast, migraine, multiple sclerosis, and tension-type headache had male-to-female ratios of less than 0·7 (table 1). In children younger than 5 years, infectious neurological conditions (ie, tetanus, meningitis, and encephalitis), particularly meningitis, were the main causes of neurological DALYs in both sexes (figure 2). DALYs from epilepsy were highest in people aged 5 years to 29 years. Migraine and tension-type headache were large contributors in young and middle-aged adults, with much higher numbers in females than males. Stroke burden rapidly increased up to the age of about 80 years, and was the dominant cause of neurological burden between ages 60 and 84 years, more so in males than females. Although Alzheimer's disease and other dementias were the dominant cause of neurological burden from age 90 years, the global number of DALYs from dementias was greatest between ages 80 and 89 years (figure 2).

**Figure 3:**
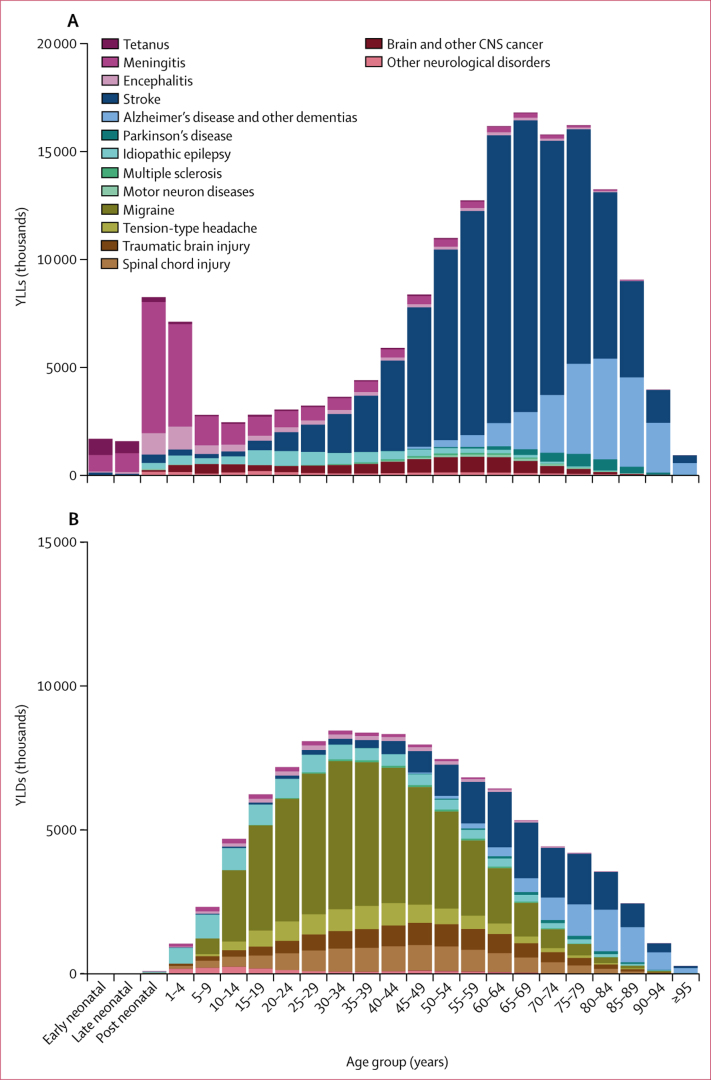
Global YLLs and YLDs for neurological disorders by age, 2016 YLLs (A) and YLDs (B). Early neonatal is 0–7 days; late neonatal is 7–28 days; post-neonatal is 28 days to 1 year. YLDs=years lived with disability. YLLs=years of life lost.

The patterns by age of YLLs and YLDs due to neurological conditions were very different. Deaths, and by extension YLLs, were the dominant feature of infectious causes, stroke, Alzheimer's disease and other dementias, and brain and other CNS cancer, whereas the burden estimated for headaches, TBI, and spinal cord injury were all YLDs, because headaches are not considered an underlying cause of death and the ICD attributes injury deaths to the cause and not the nature of injury. Epilepsy, stroke, and Alzheimer's disease and other dementias were other substantial contributors to YLDs of neurological disorders (figure 3).

Five countries had age-standardised DALY rates of combined neurological disorders exceeding 7000 DALYs per 100 000 population in 2016: Afghanistan (9135 DALYs [95% UI 7788–10 709] per 100 000), Kiribati (7477 DALYs [6596–8483] per 100 000), Solomon Islands (7104 DALYs [6075–8391] per 100 000), Central African Republic (7283 DALYs [5974–8644] per 100 000), and Somalia (7349 DALYs [5980–9416] per 100 000; table 2). All of these countries have scarce general health data, and no data for any of the neurological conditions included in this analysis. Thus, estimates depended heavily on predictive covariate values and geographical proximity and, hence, are rather uncertain. 11 countries had age-standardised DALY rates less than 2500 DALYs per 100 000 population: Singapore, Peru, Ecuador, Costa Rica, Puerto Rico, Taiwan, Japan, Australia, Switzerland, Canada, and Bermuda (table 2). The reasons for the low rates in these countries were varied. For example, Taiwan and Japan have low rates of migraine, whereas Costa Rica's stroke rates were low. High stroke rates were common among the five countries with the highest neurological DALY rates, whereas, meningitis rates were also high in Somalia, Central African Republic, and Afghanistan and tetanus rates were much higher in Somalia than in any of the other four countries. The age-standardised rates of neurological DALYs decreased significantly in 171 of 195 countries and territories between 1990 and 2016. In 23 countries, the change did not reach statistical significance, and only in North Korea was there a significant increase, which was 20% (95% UI 7–34).

**Table 2:** DALYs and age-standardised DALY rates per 100 000 people and percentage change for all neurological disorders combined by location, 1990–2016

	**Absolute numbers of DALYs (thousands)**			**Age-standardised DALY rates (per 100 000 people)**		
	1990	2016	Percentage change, 1990–2016	1990	2016	Percentage change, 1990–2016
**Global**	**240 379 (219 338 to 262 279)**	**276 143 (246 544 to 307 994)**	**15% (9 to 21)**	**5467 (5024 to 5913)**	**3968 (3557 to 4396)**	**−27% (−31 to −24)**
**High-income North America**	**8455 (7193 to 9751)**	**11 780 (10 040 to 13 527)**	**39% (37 to 42)**	**2867 (2446 to 3305)**	**2629 (2208 to 3056)**	**−8% (−10 to −7)**
Canada	794 (667 to 932)	1137 (950 to 1331)	43% (37 to 50)	2807 (2366 to 3271)	2433 (1998 to 2884)	−13% (−18 to −9)
Greenland	2 (2 to 2)	2 (1 to 2)	−12% (−24 to 1)	6956 (6236 to 7848)	4262 (3552 to 4953)	−39% (−47 to −30)
USA	7658 (6530 to 8823)	10 640 (9085 to 12 209)	39% (36 to 42)	2872 (2451 to 3310)	2652 (2232 to 3083)	−8% (−10 to −6)
**Australasia**	**624 (529 to 723)**	**860 (725 to 1007)**	**38% (32 to 44)**	**3047 (2602 to 3498)**	**2444 (2020 to 2902)**	**−20% (−24 to −15)**
Australia	516 (437 to 598)	722 (608 to 846)	40% (33 to 47)	3016 (2573 to 3482)	2430 (2002 to 2905)	−20% (−24 to −15)
New Zealand	108 (92 to 123)	138 (116 to 162)	28% (20 to 36)	3201 (2763 to 3650)	2518 (2082 to 2988)	−21% (−27 to −16)
**High-income Asia-Pacific**	**6136 (5368 to 6922)**	**7635 (6626 to 8723)**	**24% (18 to 31)**	**3643 (3210 to 4091)**	**2511 (2100 to 2959)**	**−31% (−35 to −27)**
Brunei	6 (5 to 7)	11 (9 to 13)	71% (53 to 91)	4183 (3693 to 4689)	3192 (2726 to 3738)	−24% (−31 to −16)
Japan	4336 (3781 to 4904)	5715 (4977 to 6480)	32% (27 to 37)	3202 (2786 to 3626)	2410 (2012 to 2827)	−25% (−28 to −22)
Singapore	73 (63 to 86)	102 (83 to 122)	39% (26 to 55)	3436 (2998 to 3912)	2225 (1809 to 2670)	−35% (−43 to −28)
South Korea	1730 (1506 to 1959)	1807 (1453 to 2157)	4% (−12 to 22)	5929 (5300 to 6575)	2912 (2351 to 3455)	−51% (−59 to −43)
**Western Europe**	**16 002 (13 971 to 18 071)**	**16 727 (14 215 to 19 172)**	**4% (1 to 8)**	**3489 (3004 to 3973)**	**2686 (2219 to 3174)**	**−23% (−27 to −20)**
Andorra	2 (1 to 2)	3 (3 to 4)	74% (53 to 98)	3008 (2437 to 3591)	2686 (2180 to 3231)	−11% (−21 to 0)
Austria	342 (299 to 389)	318 (264 to 375)	−7% (−13 to −1)	3651 (3139 to 4178)	2632 (2137 to 3163)	−28% (−33 to −23)
Belgium	430 (375 to 489)	454 (385 to 530)	6% (−2 to 12)	3558 (3073 to 4108)	2823 (2329 to 3363)	−21% (−27 to −15)
Cyprus	24 (21 to 27)	30 (25 to 36)	28% (19 to 37)	3663 (3169 to 4215)	2756 (2247 to 3268)	−25% (−31 to −19)
Denmark	220 (192 to 250)	206 (174 to 240)	−6% (−13 to 1)	3460 (2967 to 3962)	2693 (2232 to 3200)	−22% (−28 to −16)
Finland	227 (199 to 256)	245 (210 to 283)	8% (2 to 14)	3932 (3440 to 4459)	2995 (2498 to 3538)	−24% (−29 to −19)
France	2070 (1763 to 2376)	2349 (1985 to 2737)	13% (7 to 20)	3134 (2644 to 3638)	2576 (2101 to 3096)	−18% (−24 to −11)
Germany	3479 (3047 to 3951)	3290 (2798 to 3810)	−5% (−12 to 2)	3498 (3020 to 4025)	2645 (2172 to 3159)	−24% (−31 to −17)
Greece	488 (434 to 545)	530 (464 to 601)	9% (3 to 15)	4000 (3515 to 4505)	3002 (2535 to 3501)	−25% (−30 to −21)
Iceland	8 (7 to 10)	11 (9 to 13)	34% (27 to 42)	3229 (2748 to 3740)	2793 (2304 to 3319)	−14% (−19 to −8)
Ireland	126 (108 to 144)	145 (120 to 173)	15% (6 to 24)	3578 (3078 to 4082)	2747 (2258 to 3298)	−23% (−30 to −17)
Israel	137 (116 to 158)	223 (183 to 268)	62% (47 to 78)	3432 (2939 to 3948)	2632 (2140 to 3188)	−23% (−31 to −16)
Italy	2448 (2118 to 2794)	2684 (2279 to 3088)	10% (4 to 17)	3533 (3010 to 4084)	2729 (2228 to 3269)	−23% (−28 to −18)
Luxembourg	18 (16 to 20)	20 (16 to 23)	8% (−1 to 17)	4068 (3555 to 4624)	2704 (2221 to 3233)	−34% (−39 to −28)
Malta	12 (11 to 14)	15 (13 to 18)	22% (11 to 34)	3601 (3091 to 4140)	2700 (2187 to 3228)	−25% (−32 to −18)
Netherlands	539 (463 to 624)	614 (517 to 713)	14% (7 to 21)	3232 (2770 to 3743)	2680 (2198 to 3191)	−17% (−23 to −11)
Norway	181 (159 to 204)	181 (153 to 210)	0% (−7 to 8)	3380 (2926 to 3859)	2653 (2200 to 3134)	−22% (−27 to −15)
Portugal	604 (550 to 663)	487 (424 to 555)	−19% (−24 to −15)	5166 (4651 to 5701)	2964 (2488 to 3476)	−43% (−47 to −38)
Spain	1533 (1331 to 1744)	1791 (1511 to 2069)	17% (11 to 22)	3482 (2995 to 3982)	2596 (2118 to 3110)	−26% (−30 to −21)
Sweden	340 (297 to 385)	361 (305 to 415)	6% (−1 to 13)	2983 (2540 to 3451)	2531 (2074 to 2974)	−15% (−21 to −10)
Switzerland	266 (228 to 307)	292 (239 to 350)	10% (−1 to 23)	3244 (2744 to 3785)	2474 (1979 to 3010)	−24% (−31 to −16)
UK	2503 (2210 to 2814)	2467 (2113 to 2832)	−2% (−5 to 1)	3538 (3087 to 4032)	2771 (2303 to 3255)	−22% (−25 to −19)
**Southern Latin America**	**1702 (1509 to 1908)**	**1849 (1557 to 2150)**	**9% (1 to 16)**	**3907 (3484 to 4353)**	**2680 (2256 to 3120)**	**−31% (−36 to −27)**
Argentina	1176 (1047 to 1313)	1214 (1033 to 1411)	3% (−4 to 11)	3960 (3535 to 4408)	2696 (2288 to 3135)	−32% (−37 to −27)
Chile	396 (344 to 458)	511 (414 to 616)	29% (13 to 47)	3757 (3330 to 4254)	2592 (2101 to 3120)	−31% (−40 to −21)
Uruguay	130 (117 to 144)	124 (108 to 141)	−4% (−10 to 2)	4006 (3593 to 4464)	2927 (2500 to 3380)	−27% (−32 to −22)
**Eastern Europe**	**13 148 (11 851 to 14 406)**	**12 781 (10 708 to 15 168)**	**−3% (−16 to 12)**	**5762 (5216 to 6301)**	**4723 (3975 to 5551)**	**−18% (−28 to −6)**
Belarus	522 (467 to 579)	495 (420 to 572)	−5% (−15 to 5)	4969 (4467 to 5488)	4146 (3525 to 4771)	−17% (−25 to −9)
Estonia	92 (83 to 101)	56 (47 to 65)	−39% (−46 to −33)	5442 (4910 to 5963)	3114 (2604 to 3640)	−43% (−48 to −37)
Latvia	172 (156 to 190)	123 (106 to 138)	−29% (−34 to −22)	5881 (5336 to 6470)	4125 (3553 to 4712)	−30% (−36 to −24)
Lithuania	155 (137 to 174)	149 (132 to 167)	−4% (−9 to 2)	4167 (3678 to 4658)	3633 (3156 to 4110)	−13% (−17 to −8)
Moldova	226 (203 to 250)	201 (176 to 230)	−11% (−19 to −2)	5875 (5317 to 6431)	4492 (3944 to 5117)	−24% (−31 to −17)
Russia	8854 (7878 to 9776)	9137 (7442 to 11 332)	3% (−15 to 26)	5990 (5357 to 6584)	4972 (4087 to 6090)	−17% (−30 to 0)
Ukraine	3126 (2835 to 3440)	2622 (2169 to 3132)	−16% (−27 to −3)	5460 (4916 to 6030)	4326 (3599 to 5155)	−21% (−31 to −9)
**Central Europe**	**6289 (5663 to 6896)**	**5701 (5055 to 6380)**	**−9% (−13 to −6)**	**5172 (4678 to 5662)**	**3758 (3278 to 4241)**	**−27% (−31 to −24)**
Albania	116 (101 to 132)	140 (122 to 159)	21% (7 to 35)	4838 (4353 to 5362)	4437 (3879 to 5028)	−8% (−17 to 1)
Bosnia and Herzegovina	191 (168 to 216)	205 (179 to 235)	7% (−5 to 21)	5193 (4626 to 5763)	4137 (3593 to 4794)	−20% (−29 to −11)
Bulgaria	615 (563 to 669)	508 (441 to 572)	−17% (−26 to −8)	6120 (5564 to 6659)	4637 (3997 to 5233)	−24% (−31 to −17)
Croatia	260 (233 to 290)	225 (198 to 256)	−13% (−21 to −5)	5030 (4485 to 5606)	3662 (3173 to 4202)	−27% (−34 to −20)
Czech Republic	576 (520 to 635)	415 (355 to 479)	−28% (−33 to −23)	5201 (4686 to 5744)	3112 (2635 to 3641)	−40% (−45 to −35)
Hungary	639 (579 to 703)	467 (400 to 531)	−27% (−33 to −21)	5447 (4924 to 6007)	3496 (2976 to 4014)	−36% (−42 to −30)
Macedonia	102 (91 to 114)	124 (111 to 138)	21% (13 to 30)	6304 (5682 to 7012)	5154 (4606 to 5763)	−18% (−24 to −12)
Montenegro	30 (27 to 33)	36 (32 to 41)	21% (9 to 33)	5433 (4878 to 6006)	4772 (4218 to 5331)	−12% (−20 to −4)
Poland	1589 (1407 to 1770)	1555 (1335 to 1778)	−2% (−9 to 5)	4390 (3910 to 4872)	3246 (2782 to 3753)	−26% (−31 to −21)
Romania	1352 (1226 to 1487)	1231 (1096 to 1376)	−9% (−16 to −2)	5814 (5265 to 6371)	4492 (3961 to 5057)	−23% (−28 to −17)
Serbia	498 (440 to 559)	498 (438 to 561)	0% (−8 to 8)	5402 (4811 to 6057)	4260 (3697 to 4830)	−21% (−27 to −15)
Slovakia	225 (199 to 253)	219 (185 to 253)	−3% (−11 to 7)	4579 (4069 to 5106)	3420 (2907 to 3952)	−25% (−32 to −18)
Slovenia	88 (77 to 101)	79 (67 to 92)	−11% (−20 to −2)	4414 (3855 to 4983)	2909 (2431 to 3444)	−34% (−41 to −27)
**Central Asia**	**2740 (2477 to 3023)**	**3305 (2919 to 3726)**	**21% (15 to 27)**	**5522 (5055 to 6014)**	**4661 (4197 to 5173)**	**−16% (−19 to −12)**
Armenia	117 (102 to 135)	105 (89 to 122)	−11% (−18 to −3)	4325 (3839 to 4844)	3184 (2714 to 3713)	−26% (−32 to −20)
Azerbaijan	247 (216 to 280)	359 (302 to 420)	45% (27 to 63)	4924 (4415 to 5463)	4248 (3627 to 4917)	−14% (−24 to −4)
Georgia	306 (276 to 338)	228 (199 to 261)	−25% (−34 to −16)	5721 (5177 to 6290)	4543 (3947 to 5206)	−21% (−29 to −11)
Kazakhstan	703 (625 to 791)	694 (589 to 809)	−1% (−12 to 12)	5586 (5029 to 6189)	4614 (3941 to 5317)	−17% (−26 to −7)
Kyrgyzstan	198 (179 to 218)	207 (181 to 235)	5% (−4 to 14)	6426 (5918 to 6973)	4867 (4364 to 5438)	−24% (−30 to −18)
Mongolia	95 (76 to 116)	141 (123 to 162)	50% (21 to 85)	5749 (5075 to 6485)	6460 (5729 to 7287)	13% (−2 to 29)
Tajikistan	213 (185 to 242)	279 (238 to 326)	31% (12 to 53)	5819 (5211 to 6476)	5037 (4427 to 5718)	−13% (−23 to −2)
Turkmenistan	137 (122 to 153)	206 (181 to 232)	50% (37 to 65)	5646 (5164 to 6181)	5085 (4582 to 5587)	−10% (−16 to −3)
Uzbekistan	722 (637 to 820)	1083 (922 to 1262)	50% (34 to 67)	5300 (4761 to 5896)	4631 (4027 to 5309)	−13% (−21 to −4)
**Central Latin America**	**4147 (3576 to 4743)**	**5654 (4688 to 6652)**	**36% (29 to 43)**	**3410 (3016 to 3836)**	**2631 (2241 to 3048)**	**−23% (−26 to −19)**
Colombia	894 (758 to 1053)	1078 (875 to 1309)	21% (6 to 38)	3692 (3246 to 4213)	2542 (2119 to 3035)	−31% (−38 to −23)
Costa Rica	62 (51 to 75)	107 (87 to 130)	72% (53 to 92)	2799 (2395 to 3241)	2336 (1937 to 2803)	−17% (−25 to −8)
El Salvador	161 (138 to 185)	140 (114 to 168)	−13% (−23 to −1)	4033 (3536 to 4548)	2538 (2106 to 3000)	−37% (−44 to −30)
Guatemala	220 (188 to 255)	346 (280 to 419)	58% (35 to 80)	3314 (2843 to 3777)	2823 (2315 to 3365)	−15% (−27 to −2)
Honduras	200 (173 to 228)	224 (185 to 269)	13% (−5 to 36)	4829 (4234 to 5470)	3461 (2887 to 4126)	−28% (−39 to −14)
Mexico	1979 (1695 to 2284)	2800 (2332 to 3313)	41% (35 to 48)	3158 (2755 to 3576)	2562 (2173 to 2977)	−19% (−22 to −16)
Nicaragua	101 (85 to 120)	128 (103 to 155)	27% (10 to 47)	3500 (3026 to 3996)	2644 (2195 to 3120)	−24% (−33 to −15)
Panama	61 (53 to 71)	94 (78 to 113)	55% (38 to 74)	3526 (3122 to 3997)	2681 (2256 to 3161)	−24% (−32 to −16)
Venezuela	467 (394 to 554)	732 (592 to 885)	57% (35 to 81)	3623 (3180 to 4140)	2837 (2350 to 3390)	−22% (−31 to −11)
**Andean Latin America**	**1114 (981 to 1258)**	**1248 (1037 to 1496)**	**12% (1 to 25)**	**3715 (3321 to 4148)**	**2457 (2073 to 2913)**	**−34% (−40 to −28)**
Bolivia	272 (234 to 310)	272 (225 to 322)	0% (−16 to 17)	4942 (4339 to 5634)	3022 (2517 to 3552)	−39% (−47 to −30)
Ecuador	267 (229 to 312)	346 (282 to 416)	29% (13 to 49)	3529 (3096 to 4029)	2467 (2061 to 2928)	−30% (−38 to −21)
Peru	575 (493 to 657)	630 (509 to 778)	10% (−5 to 28)	3411 (2964 to 3835)	2271 (1868 to 2742)	−33% (−41 to −24)
**Caribbean**	**1576 (1383 to 1786)**	**1544 (1346 to 1784)**	**−2% (−14 to 9)**	**5057 (4518 to 5636)**	**3539 (3108 to 4056)**	**−30% (−37 to −23)**
Antigua and Barbuda	2 (2 to 2)	3 (2 to 3)	16% (3 to 30)	4249 (3780 to 4758)	2987 (2538 to 3492)	−30% (−37 to −22)
The Bahamas	7 (6 to 8)	12 (10 to 14)	68% (51 to 85)	3909 (3470 to 4375)	3142 (2683 to 3604)	−20% (−27 to −12)
Barbados	10 (8 to 11)	10 (9 to 12)	6% (−2 to 16)	3853 (3421 to 4321)	2963 (2555 to 3428)	−23% (−29 to −16)
Belize	5 (4 to 6)	8 (7 to 9)	63% (43 to 85)	3606 (3206 to 4065)	3253 (2830 to 3719)	−10% (−18 to −1)
Bermuda	2 (1 to 2)	2 (1 to 2)	2% (−9 to 14)	3980 (3514 to 4491)	2492 (2081 to 2945)	−37% (−44 to −30)
Cuba	341 (301 to 385)	395 (342 to 449)	16% (8 to 25)	3542 (3159 to 3965)	2795 (2394 to 3202)	−21% (−27 to −15)
Dominica	2 (2 to 2)	2 (2 to 3)	15% (3 to 30)	3673 (3258 to 4145)	3155 (2712 to 3645)	−14% (−22 to −4)
Dominican Republic	215 (188 to 246)	267 (222 to 313)	24% (8 to 40)	4168 (3716 to 4650)	3049 (2590 to 3540)	−27% (−35 to −19)
Grenada	4 (3 to 4)	4 (3 to 4)	−4% (−15 to 9)	4892 (4404 to 5433)	3881 (3384 to 4413)	−21% (−30 to −11)
Guyana	33 (30 to 36)	29 (25 to 32)	−13% (−23 to −3)	7493 (6942 to 8090)	4876 (4350 to 5450)	−35% (−41 to −28)
Haiti	698 (566 to 867)	513 (414 to 642)	−26% (−43 to −7)	10 731 (9117 to 12 733)	6137 (5102 to 7235)	−43% (−53 to −31)
Jamaica	86 (76 to 98)	98 (84 to 114)	14% (0 to 28)	4332 (3848 to 4831)	3584 (3077 to 4127)	−17% (−27 to −8)
Puerto Rico	89 (76 to 105)	103 (87 to 121)	16% (7 to 27)	2696 (2305 to 3147)	2359 (1988 to 2789)	−12% (−20 to −4)
Saint Lucia	5 (4 to 5)	6 (5 to 7)	17% (6 to 29)	4503 (4053 to 4964)	3139 (2723 to 3611)	−30% (−36 to −24)
Saint Vincent and the Grenadines	3 (3 to 4)	4 (3 to 4)	9% (−2 to 20)	4266 (3816 to 4750)	3758 (3307 to 4247)	−12% (−19 to −4)
Suriname	15 (13 to 17)	20 (18 to 23)	37% (23 to 51)	4726 (4287 to 5218)	4318 (3845 to 4832)	−9% (−16 to −1)
Trinidad and Tobago	42 (37 to 47)	44 (38 to 51)	7% (−3 to 18)	4660 (4220 to 5122)	3310 (2880 to 3815)	−29% (−35 to −22)
Virgin Islands	3 (2 to 3)	4 (3 to 4)	43% (28 to 59)	3221 (2819 to 3705)	2752 (2328 to 3235)	−15% (−23 to −6)
**Tropical Latin America**	**5303 (4757 to 5894)**	**6470 (5604 to 7378)**	**22% (16 to 27)**	**5185 (4768 to 5648)**	**3345 (2941 to 3761)**	**−36% (−39 to −32)**
Brazil	5188 (4654 to 5761)	6288 (5443 to 7172)	21% (15 to 27)	5215 (4789 to 5681)	3341 (2939 to 3757)	−36% (−39 to −33)
Paraguay	114 (99 to 130)	181 (154 to 211)	59% (43 to 76)	4117 (3681 to 4567)	3541 (3074 to 4048)	−14% (−22 to −5)
**East Asia**	**51 041 (47 231 to 55 200)**	**61 366 (55 894 to 66 880)**	**20% (13 to 26)**	**6205 (5792 to 6702)**	**4115 (3752 to 4475)**	**−34% (−38 to −30)**
China	49 814 (46 115 to 53 839)	59 292 (54 070 to 64 582)	19% (12 to 25)	6273 (5853 to 6780)	4126 (3776 to 4497)	−34% (−39 to −30)
North Korea	646 (560 to 730)	1402 (1256 to 1555)	118% (94 to 142)	4663 (4091 to 5222)	5569 (5018 to 6138)	20% (7 to 34)
Taiwan (Province of China)	581 (514 to 651)	675 (575 to 785)	16% (6 to 27)	4011 (3635 to 4408)	2382 (2035 to 2761)	−41% (−46 to −35)
**Southeast Asia**	**17 191 (15 142 to 19 147)**	**22 694 (20 351 to 25 135)**	**32% (22 to 42)**	**5422 (4944 to 5880)**	**4293 (3910 to 4691)**	**−21% (−25 to −16)**
Cambodia	769 (575 to 1096)	541 (476 to 613)	−28% (−53 to −4)	11 005 (8966 to 14 491)	5363 (4850 to 5922)	−51% (−63 to −40)
Indonesia	6849 (5419 to 8075)	9260 (8270 to 10 280)	36% (18 to 63)	5295 (4613 to 5961)	4785 (4345 to 5229)	−9% (−18 to 0)
Laos	338 (256 to 470)	263 (221 to 317)	−20% (−46 to 6)	9753 (8128 to 12 675)	5511 (4862 to 6127)	−43% (−57 to −31)
Malaysia	516 (455 to 580)	806 (691 to 928)	56% (42 to 70)	4911 (4499 to 5356)	3456 (3035 to 3873)	−30% (−35 to −25)
Maldives	6 (5 to 7)	7 (5 to 8)	9% (−11 to 30)	4917 (4312 to 5586)	2605 (2157 to 3116)	−47% (−55 to −38)
Mauritius	37 (33 to 41)	40 (35 to 47)	9% (−2 to 21)	5367 (4940 to 5809)	3151 (2717 to 3635)	−41% (−47 to −35)
Myanmar	1913 (1648 to 2191)	2004 (1754 to 2303)	5% (−10 to 22)	7118 (6318 to 8037)	4964 (4438 to 5592)	−30% (−39 to −21)
Philippines	1779 (1585 to 2002)	3318 (2887 to 3780)	87% (69 to 107)	4486 (4078 to 4924)	4614 (4073 to 5176)	3% (−7 to 14)
Sri Lanka	464 (398 to 534)	598 (493 to 713)	29% (13 to 48)	3720 (3254 to 4175)	3088 (2573 to 3625)	−17% (−27 to −6)
Seychelles	3 (2 to 3)	3 (3 to 3)	15% (1 to 30)	4584 (4064 to 5100)	3279 (2846 to 3784)	−28% (−36 to −20)
Thailand	1611 (1405 to 1837)	2149 (1829 to 2487)	33% (22 to 47)	4080 (3657 to 4524)	2936 (2517 to 3398)	−28% (−34 to −21)
Timor-Leste	38 (28 to 52)	32 (26 to 39)	−13% (−41 to 17)	7358 (5973 to 10 288)	4356 (3547 to 5126)	−40% (−60 to −24)
Vietnam	2855 (2475 to 3286)	3643 (3217 to 4120)	28% (11 to 47)	6094 (5347 to 6967)	4403 (3930 to 4929)	−28% (−37 to −17)
**Oceania**	**278 (241 to 318)**	**453 (385 to 528)**	**64% (40 to 91)**	**6675 (5920 to 7501)**	**5708 (4950 to 6484)**	**−14% (−25 to −2)**
American Samoa	1 (1 to 1)	2 (2 to 2)	48% (28 to 68)	4475 (4013 to 4954)	3591 (3104 to 4107)	−20% (−29 to −9)
Federated States of Micronesia	4 (3 to 4)	4 (3 to 5)	4% (−17 to 28)	6909 (5992 to 7925)	5898 (4870 to 7095)	−14% (−31 to 4)
Fiji	20 (17 to 24)	29 (24 to 34)	42% (13 to 77)	4706 (3919 to 5562)	4149 (3461 to 4885)	−11% (−29 to 9)
Guam	3 (3 to 3)	5 (5 to 6)	86% (67 to 108)	3497 (3090 to 3897)	3387 (2947 to 3860)	−3% (−12 to 7)
Kiribati	4 (4 to 5)	6 (5 to 7)	37% (14 to 65)	8312 (7508 to 9152)	7477 (6596 to 8483)	−10% (−22 to 4)
Marshall Islands	1 (1 to 2)	2 (2 to 3)	56% (33 to 80)	5425 (4834 to 5998)	4648 (4024 to 5336)	−14% (−25 to −2)
Northern Mariana Islands	1 (1 to 1)	2 (2 to 3)	134% (95 to 177)	4201 (3571 to 4858)	3300 (2815 to 3790)	−21% (−34 to −7)
Papua New Guinea	211 (179 to 249)	349 (290 to 420)	66% (36 to 102)	8172 (7063 to 9467)	6825 (5749 to 7933)	−16% (−29 to −1)
Samoa	5 (4 to 6)	6 (5 to 7)	16% (−1 to 32)	5335 (4702 to 6048)	4235 (3630 to 4839)	−20% (−31 to −10)
Solomon Islands	12 (10 to 15)	25 (21 to 29)	101% (69 to 142)	7860 (6720 to 9116)	7104 (6075 to 8391)	−9% (−23 to 8)
Tonga	3 (2 to 4)	3 (3 to 4)	7% (−13 to 29)	4363 (3789 to 5000)	3793 (3287 to 4300)	−13% (−25 to 1)
Vanuatu	6 (5 to 7)	12 (10 to 14)	99% (67 to 138)	7380 (6333 to 8453)	6726 (5726 to 7972)	−9% (−22 to 8)
**North Africa and Middle East**	**12 521 (10 765 to 14 400)**	**17 300 (14 801 to 20 108)**	**38% (25 to 51)**	**5033 (4483 to 5597)**	**4048 (3534 to 4593)**	**−20% (−24 to −15)**
Afghanistan	1440 (1024 to 2063)	1923 (1549 to 2457)	37% (−7 to 88)	11 896 (9796 to 14 634)	9135 (7788 to 10 709)	−23% (−36 to −10)
Algeria	683 (564 to 810)	1080 (892 to 1280)	58% (40 to 77)	4239 (3660 to 4821)	3410 (2899 to 3953)	−20% (−26 to −13)
Bahrain	11 (9 to 13)	27 (20 to 33)	148% (118 to 180)	3788 (3253 to 4330)	2841 (2340 to 3377)	−25% (−33 to −16)
Egypt	2065 (1814 to 2328)	2790 (2331 to 3291)	35% (20 to 50)	5105 (4551 to 5697)	4204 (3607 to 4868)	−18% (−25 to −9)
Iran	1474 (1197 to 1787)	2244 (1816 to 2732)	53% (33 to 75)	4255 (3646 to 4909)	3601 (3005 to 4220)	−15% (−25 to −4)
Iraq	773 (607 to 984)	1335 (1068 to 1668)	74% (44 to 108)	6556 (5486 to 7823)	5711 (4692 to 6862)	−13% (−25 to 1)
Jordan	80 (67 to 94)	166 (132 to 203)	107% (80 to 137)	4463 (3903 to 5044)	3410 (2835 to 4053)	−24% (−35 to −12)
Kuwait	35 (27 to 44)	76 (57 to 97)	118% (94 to 145)	2999 (2532 to 3548)	3033 (2443 to 3642)	1% (−12 to 15)
Lebanon	106 (75 to 150)	172 (122 to 239)	63% (44 to 87)	4910 (3743 to 6453)	3219 (2421 to 4359)	−34% (−42 to −27)
Libya	119 (98 to 146)	168 (136 to 205)	42% (19 to 65)	4050 (3501 to 4694)	3657 (3084 to 4280)	−10% (−20 to 1)
Morocco	991 (792 to 1210)	1023 (848 to 1216)	4% (−14 to 23)	4934 (4237 to 5684)	3643 (3114 to 4246)	−26% (−35 to −18)
Palestine	64 (50 to 78)	132 (110 to 158)	108% (74 to 149)	5366 (4612 to 6160)	5245 (4642 to 5888)	−2% (−12 to 9)
Oman	73 (58 to 91)	97 (77 to 119)	35% (0 to 72)	4884 (4235 to 5577)	3149 (2699 to 3626)	−35% (−43 to −26)
Qatar	11 (9 to 13)	40 (30 to 51)	272% (219 to 330)	3622 (3068 to 4223)	2643 (2124 to 3215)	−27% (−38 to −15)
Saudi Arabia	358 (296 to 424)	695 (559 to 840)	94% (78 to 112)	4133 (3574 to 4701)	3569 (3090 to 4081)	−14% (−21 to −6)
Sudan	800 (669 to 961)	1141 (943 to 1352)	43% (23 to 64)	5996 (5197 to 6950)	4513 (3907 to 5170)	−25% (−32 to −18)
Syria	433 (371 to 505)	499 (398 to 618)	15% (−6 to 37)	5065 (4514 to 5636)	3830 (3206 to 4582)	−24% (−33 to −15)
Tunisia	228 (193 to 267)	333 (273 to 394)	46% (30 to 64)	3992 (3466 to 4572)	3287 (2748 to 3849)	−18% (−26 to −9)
Turkey	2203 (1647 to 2782)	2302 (1891 to 2722)	6% (−16 to 39)	4665 (3760 to 5597)	3245 (2709 to 3788)	−30% (−41 to −15)
United Arab Emirates	49 (39 to 60)	251 (204 to 306)	415% (321 to 516)	4988 (4225 to 5937)	4025 (3461 to 4681)	−19% (−33 to −4)
Yemen	511 (417 to 646)	776 (650 to 911)	53% (23 to 86)	6744 (5797 to 7782)	5157 (4468 to 5856)	−23% (−32 to −14)
**South Asia**	**56 902 (49 124 to 64 150)**	**56 158 (49 114 to 63 659)**	**−1% (−13 to 13)**	**6103 (5459 to 6731)**	**4193 (3768 to 4660)**	**−31% (−38 to −25)**
Bangladesh	5569 (4106 to 6565)	5549 (4751 to 6369)	1% (−17 to 33)	7063 (6076 to 7857)	4646 (4090 to 5217)	−34% (−42 to −24)
Bhutan	32 (23 to 43)	22 (18 to 26)	−30% (−51 to −8)	6606 (5436 to 8048)	3552 (3035 to 4108)	−46% (−57 to −36)
India	43 351 (37 483 to 49 294)	42 665 (37 236 to 48 655)	−1% (−14 to 13)	5912 (5244 to 6557)	4029 (3589 to 4500)	−32% (−38 to −25)
Nepal	1708 (1150 to 2543)	955 (797 to 1132)	−42% (−62 to −20)	8424 (6611 to 10 789)	4217 (3628 to 4842)	−49% (−61 to −38)
Pakistan	6240 (5281 to 7377)	6958 (5932 to 8175)	12% (−9 to 38)	6415 (5671 to 7237)	5034 (4410 to 5746)	−21% (−32 to −9)
**Southern sub-Saharan Africa**	**1503 (1327 to 1680)**	**2298 (2048 to 2577)**	**53% (43 to 64)**	**4091 (3662 to 4498)**	**3939 (3574 to 4330)**	**−4% (−10 to 3)**
Botswana	34 (28 to 40)	60 (41 to 79)	77% (25 to 129)	4752 (3945 to 5594)	4064 (2676 to 5292)	−14% (−42 to 12)
Lesotho	50 (43 to 58)	77 (63 to 94)	54% (25 to 89)	5230 (4467 to 6024)	6010 (4831 to 7378)	15% (−8 to 43)
Namibia	46 (40 to 53)	61 (46 to 75)	32% (4 to 59)	5901 (5271 to 6590)	3989 (3028 to 4827)	−32% (−47 to −19)
South Africa	1098 (966 to 1235)	1535 (1353 to 1735)	40% (30 to 49)	4019 (3615 to 4436)	3658 (3285 to 4072)	−9% (−14 to −3)
Swaziland	24 (20 to 28)	37 (28 to 47)	59% (28 to 94)	5304 (4559 to 6144)	4611 (3412 to 5928)	−13% (−32 to 9)
Zimbabwe	250 (199 to 299)	527 (443 to 619)	112% (74 to 167)	3926 (2959 to 4644)	4708 (3957 to 5516)	21% (−1 to 59)
**Western sub-Saharan Africa**	**12 407 (10 421 to 14 457)**	**14 438 (12 137 to 17 921)**	**17% (−2 to 43)**	**6383 (5667 to 7160)**	**4593 (4069 to 5283)**	**−28% (−35 to −19)**
Benin	331 (272 to 405)	404 (338 to 504)	23% (−5 to 59)	6792 (6005 to 7717)	5077 (4472 to 5836)	−25% (−35 to −13)
Burkina Faso	708 (540 to 919)	753 (593 to 984)	8% (−21 to 47)	6909 (5850 to 8250)	4950 (4288 to 5864)	−28% (−40 to −14)
Cameroon	590 (484 to 741)	904 (743 to 1104)	55% (21 to 94)	5672 (4947 to 6584)	5080 (4239 to 6031)	−10% (−24 to 5)
Cape Verde	12 (10 to 14)	14 (11 to 16)	16% (−5 to 39)	4611 (4045 to 5223)	3332 (2825 to 3919)	−28% (−37 to −17)
Chad	468 (367 to 645)	715 (582 to 887)	55% (4 to 102)	7412 (6314 to 9394)	5615 (4893 to 6518)	−24% (−39 to −10)
Côte d'Ivoire	614 (504 to 767)	877 (746 to 1037)	44% (15 to 74)	6351 (5591 to 7151)	5587 (4919 to 6332)	−12% (−22 to −1)
The Gambia	41 (34 to 49)	62 (50 to 80)	51% (21 to 90)	5289 (4619 to 6041)	4396 (3764 to 5170)	−17% (−27 to −5)
Ghana	806 (638 to 1040)	994 (837 to 1181)	25% (−4 to 54)	7023 (6048 to 8128)	5295 (4588 to 5998)	−24% (−35 to −13)
Guinea	523 (409 to 669)	554 (466 to 662)	8% (−22 to 40)	8021 (6821 to 9475)	5716 (5025 to 6495)	−28% (−41 to −15)
Guinea-Bissau	91 (74 to 111)	95 (78 to 124)	5% (−20 to 42)	9285 (8092 to 10 686)	6834 (5896 to 8044)	−26% (−38 to −13)
Liberia	144 (117 to 182)	151 (124 to 199)	6% (−20 to 38)	6374 (5514 to 7319)	4774 (4159 to 5480)	−25% (−36 to −14)
Mali	740 (584 to 988)	724 (562 to 1023)	−1% (−28 to 32)	8601 (7195 to 11 257)	5078 (4299 to 6139)	−40% (−55 to −27)
Mauritania	83 (68 to 111)	116 (92 to 145)	41% (9 to 77)	5869 (4959 to 7561)	4025 (3274 to 4899)	−31% (−44 to −17)
Niger	1206 (842 to 1637)	1034 (799 to 1400)	−12% (−42 to 32)	10 905 (8469 to 14 054)	5690 (4730 to 6878)	−47% (−60 to −32)
Nigeria	5169 (4160 to 6399)	5931 (4606 to 8011)	16% (−13 to 58)	5493 (4743 to 6341)	3701 (3088 to 4494)	−32% (−44 to −18)
São Tomé and Príncipe	5 (4 to 6)	5 (4 to 6)	9% (−13 to 34)	5152 (4512 to 5833)	4219 (3562 to 4882)	−18% (−31 to −5)
Senegal	390 (327 to 469)	550 (453 to 724)	42% (14 to 84)	5984 (5293 to 6774)	5091 (4396 to 6048)	−15% (−24 to −3)
Sierra Leone	306 (245 to 397)	302 (241 to 427)	0% (−25 to 33)	7515 (6404 to 8993)	5859 (4995 to 7162)	−22% (−34 to −7)
Togo	181 (150 to 219)	250 (211 to 297)	39% (14 to 65)	5984 (5222 to 6790)	5077 (4429 to 5818)	−15% (−25 to −4)
**Eastern sub-Saharan Africa**	**13 271 (11 356 to 15 291)**	**13 325 (11 696 to 15 108)**	**1% (−14 to 20)**	**8046 (7185 to 9246)**	**4896 (4419 to 5418)**	**−39% (−45 to −33)**
Burundi	431 (341 to 556)	444 (366 to 542)	5% (−23 to 38)	10 205 (8580 to 12 443)	5467 (4703 to 6325)	−46% (−57 to −35)
Comoros	23 (19 to 29)	23 (19 to 27)	−3% (−23 to 21)	7376 (6354 to 8679)	4265 (3698 to 4919)	−42% (−51 to −33)
Djibouti	32 (25 to 41)	32 (25 to 40)	2% (−24 to 35)	6091 (5147 to 7318)	4282 (3482 to 5116)	−29% (−42 to −15)
Eritrea	247 (178 to 343)	185 (152 to 225)	−23% (−47 to 3)	10 360 (8331 to 13 225)	5499 (4681 to 6404)	−46% (−57 to −36)
Ethiopia	3699 (2871 to 4822)	3283 (2765 to 3889)	−10% (−34 to 18)	9248 (7840 to 11 226)	4774 (4124 to 5476)	−48% (−58 to −37)
Kenya	1321 (1034 to 1907)	1337 (1136 to 1593)	3% (−19 to 22)	6058 (4646 to 8720)	4047 (3417 to 4813)	−32% (−47 to −22)
Madagascar	675 (581 to 777)	1036 (867 to 1229)	54% (28 to 82)	7997 (7156 to 9041)	6492 (5425 to 7716)	−19% (−31 to −6)
Malawi	677 (517 to 873)	590 (476 to 737)	−11% (−39 to 26)	6906 (5700 to 8183)	4428 (3663 to 5244)	−35% (−49 to −20)
Mozambique	947 (772 to 1187)	984 (817 to 1201)	5% (−21 to 36)	8366 (7432 to 9403)	5029 (4269 to 5871)	−40% (−49 to −29)
Rwanda	530 (409 to 687)	385 (312 to 480)	−26% (−45 to −2)	8780 (7461 to 10 314)	4507 (3721 to 5433)	−48% (−59 to −37)
Somalia	673 (452 to 1073)	558 (425 to 781)	−15% (−40 to 13)	12 002 (8731 to 18 830)	7349 (5980 to 9416)	−37% (−54 to −22)
South Sudan	727 (471 to 1147)	778 (580 to 1076)	11% (−26 to 62)	9433 (6933 to 15 320)	5981 (4806 to 7593)	−35% (−54 to −16)
Tanzania	1247 (1046 to 1450)	1631 (1333 to 2021)	32% (5 to 69)	5741 (5013 to 6519)	4170 (3544 to 4799)	−27% (−37 to −16)
Uganda	1361 (1099 to 1783)	1416 (1158 to 1788)	5% (−24 to 40)	7897 (6902 to 9100)	4812 (4158 to 5580)	−39% (−49 to −29)
Zambia	678 (520 to 854)	633 (521 to 769)	−5% (−32 to 31)	7424 (6152 to 8706)	5462 (4542 to 6532)	−26% (−42 to −7)
**Central sub-Saharan Africa**	**2600 (2143 to 3100)**	**3907 (3312 to 4716)**	**51% (21 to 92)**	**6485 (5695 to 7385)**	**5107 (4559 to 5697)**	**−21% (−30 to −12)**
Angola	637 (494 to 815)	756 (606 to 941)	20% (−11 to 63)	7313 (5866 to 9162)	4809 (3976 to 5852)	−34% (−49 to −17)
Central African Republic	199 (164 to 242)	263 (214 to 319)	33% (3 to 63)	8458 (7221 to 9841)	7283 (5974 to 8644)	−14% (−29 to 2)
Congo (Brazzaville)	111 (93 to 137)	145 (118 to 177)	31% (5 to 63)	7054 (6028 to 8241)	4790 (4005 to 5601)	−32% (−44 to −19)
Democratic Republic of the Congo	1581 (1219 to 2001)	2672 (2183 to 3394)	72% (27 to 139)	5985 (5151 to 6856)	5107 (4468 to 5719)	−14% (−25 to −2)
Equatorial Guinea	27 (22 to 35)	18 (14 to 24)	−32% (−53 to −6)	8578 (7081 to 10 528)	3346 (2494 to 4320)	−61% (−72 to −48)
Gabon	44 (38 to 52)	53 (44 to 64)	20% (−4 to 47)	5828 (5071 to 6679)	4154 (3498 to 4846)	−29% (−40 to −16)

Numbers in brackets are 95% uncertainty intervals. DALY=disability-adjusted life-year.

Only three of the 15 neurological disorder categories had more than 10% of DALYs attributable to the 84 risks quantified in GBD 2016: risk-attributable DALYs accounted for 88·8% (86·5–90·9) of all stroke DALYs, 22·3% (11·8–35·1) of DALYs for Alzheimer's disease and other dementias, and 14·1% (10·8–17·5) of idiopathic epilepsy DALYs. For the other neurological disorders, the proportion of DALYs that were risk attributable was either very small (meningitis, encephalitis, and multiple sclerosis) or zero (tetanus, brain and other CNS cancer, migraine and tension-type headache, Parkinson's disease, and motor neuron diseases).

## Discussion

Contributing 11·6% of global DALYs and 16·5% of deaths from all causes, neurological disorders remain the leading group cause of DALYs and the second leading group cause of deaths in the world. These estimates are close to those reported in GBD 2015 estimates on the global burden of neurological disorders. Furthermore, our findings reported in the individual neurological disorder papers of the significant geographic and sex variations and the effects of SDI on the patterns of the burden of neurological disorders were consistent with our previous observations.^2^ Although the age-standardised rates of deaths and DALYs from 1990 to 2016 have decreased significantly, the absolute numbers of deaths and DALYs have significantly increased over the same period. The only neurological disorders that had significant decreases in age-standardised incidence, prevalence, mortality, and DALY rates were infectious neurological disorders (meningitis, encephalitis, and tetanus). These diverging trends in communicable and non-communicable neurological disorders are in line with our previous estimates^2^ and consistent with the observed overall global burden shift from communicable to non-communicable disorders.^18^ The decline of age-standardised rates with concomitant increase in absolute numbers was observed in most countries. The increase in absolute numbers of non-communicable neurological disorders juxtaposed with a significant decrease in their age-standardised rates is consistent with the major role of population ageing and growth as the main drivers of this increase; it also suggests that intervention strategies to reduce non-communicable neurological disorders are not sufficiently deployed or effective. With continuing ageing of populations, the prevalence of non-communicable neurological disorders is likely to continue increasing, placing pressure on already overstretched health-care services.^19^, ^20^, ^21^ Therefore, development of new strategies to treat or prevent the major neurological disorders and the implementation of already proven effective prevention strategies for stroke and infectious neurological disorders are of paramount importance to achieve UN Sustainable Development Goal targets.

The relationship between SDI and age-standardised DALY rates has been discussed in each of the neurological disorder-specific GBD 2016 papers.^3^, ^4^, ^5^, ^6^, ^7^, ^8^, ^9^, ^10^, ^11^, ^12^ For stroke, epilepsy, and meningitis we showed that age-standardised DALY rates by GBD regions over the period 1990–2016 follow a downward trajectory aligned with the predicted direction of decreasing burden of these conditions with socioeconomic development. Four other categories, brain and other CNS cancer, Parkinson's disease, multiple sclerosis, and motor neuron diseases, appear to follow a pattern of increasing DALY rates with increasing SDI. However, for all four of these conditions there were large variations in rates from the expected pattern according to SDI, and high-income Asia Pacific has much lower rates for all four conditions than other high-income regions. This suggests that the observed relationship with SDI is spurious, with SDI being a proxy for other factors (eg, genetic) explaining variations in disease rates. For Alzheimer's disease and other dementias and migraine and tension-type headache, the variation in age-standardised DALY rates and SDI between world regions and within regions over time suggests that socioeconomic development does not determine the extent of DALYs from these diseases.

Our study findings have important health service implications. The increase in number of people affected by non-communicable neurological disorders implies a need for substantially increased resources for their management. Our detailed estimates for 195 countries and a growing number of subnational locations provides a basis for locality-specific priority setting and financing of health services, including workforce development. For example, by knowing the absolute numbers of new and existing cases of major disabling neurological disorders from the GBD study, one can estimate annual requirements for first specialist neurologist assessments (total number of incident cases of these disorders) and for follow-up neurological assessments (assuming an average number [eg, two] of follow-up visits per year), and from these, the number of neurologists required in a country for outpatient management. For example, in New Zealand in 2016, there were 23 132 incident and 152 210 prevalent cases of stroke, dementias, refractory migraines (perhaps 5% of all migraine cases),^22^ epilepsy, Parkinson's disease, multiple sclerosis, motor neuron diseases, and brain and other CNS cancers combined. Thus, the total time for required first specialist assessments (23 132 × 0·75 h for a new clinic patient as reported in New Zealand^23^ is 17 349 h) and neurology follow-ups (152 210 [number of prevalent cases] × two [average number of follow-ups per year] × 0·5 h for a follow-up is 152 210 h) was 169 559 h. By dividing 169 559 h by the average estimate of physicians' working hours per year (40 h per week × 48 weeks is 1920 h), one can estimate that approximately 88 full-time equivalent (FTE) neurologists are required (compared with the 36 that are available)^23^ to provide specialist neurological care for the most common disabling neurological disorders in New Zealand. This result is close to previous estimates of FTE neurologist requirements in New Zealand using a pragmatic analysis.^23^

This study was not free of the general limitations shared by all GBD estimates. First, to bring as much information as possible into the estimation of disease and death rates for GBD, an extensive effort is dedicated to making data sources comparable—eg, by mapping cause-of-death data across various iterations of ICD classification systems and the local idiosyncrasies in how these are applied; the redistribution of less informative codes for an underlying cause of death to specified causes in the GBD list; and adjustments of non-fatal and risk factor data to account for biases introduced by different case definitions or study methods. However, we cannot guarantee that we are aware of and can accurately correct for all sources of measurement bias, particularly for diseases like dementia, multiple sclerosis, motor neuron diseases, and Parkinson's disease, which require more complex survey procedures to establish a diagnosis. Additionally, diagnosis of headaches is entirely based on patient report and thus, prone to various sources of reporting bias.^24^ Second, despite extensive efforts to identify data sources for GBD, many countries have sparse data and missing information. In those countries, we rely heavily on predictive covariates and assume similarity through geographical proximity. However, many of the neurological disorders do not have strong predictors. The exception is stroke, for which the combination of all risks included in GBD predicts 88·8% of the total burden.^12^ The absence of strong predictors in the models of the other disorders makes it much harder to predict disease levels in countries with sparse or no data. It also makes it more essential to conduct good-quality epidemiological studies in those countries that are currently poorly represented in the disease models. Third, we were able to add spinal cord injury and TBI estimates to this analysis but only in terms of incidence and YLDs. The dominant tradition in cause-of-death attribution is to assign injury deaths to the cause of the injury rather than the consequent nature of injury. In future iterations of GBD we plan to analyse data sources with multiple causes listed for each death to approximate deaths from nature of injury categories like TBI and spinal cord injury. Fourth, in GBD, the correction for comorbidity is made on the basis of the independent probability that an individual will have combinations of disabling sequelae. Although we would like also to take into account the more commonly known comorbidities, computationally this has remained intractable for the large number of diseases and their sequelae estimated in GBD. For the neurological disorders that are highly prevalent in older people with known comorbidities such as stroke and diabetes, we are overestimating the non-fatal burden. Finally, the residual category of other neurological disorders tries to capture deaths and DALYs for neurological disorders that are not explicitly being estimated. For deaths, where vital registration systems provide data on all causes, this can be estimated with greater precision. For the non-fatal estimates, we approximate YLDs by assuming the same ratio of YLDs and YLLs estimated for the main fatal neurological disorders (Alzheimer's disease and other dementias, Parkinson's disease, multiple sclerosis, motor neuron diseases, and idiopathic epilepsy) and apply this ratio to the YLLs calculated for residual causes like Huntington's disease and muscular dystrophy. Although as a placeholder estimate this method has at least some validity for residual neurological disorders that lead to death, it does not capture the burden of any residual neurological disorders that are not a cause of death, such as peripheral neuropathies, neuropathic pain, or restless legs syndrome. Thus, our estimates of the burden of neurological disorders, although already large and increasing, are still an underestimate of the true burden of neurological disorders and will be until resources are found to explicitly estimate the burden of these residual disorders.

In summary, the burden of neurological disorders (especially non-communicable disorders) is large and increasing, posing a challenge to the sustainability of health systems. Although a wealth of knowledge is available on how best to reduce the burden of stroke and infectious neurological diseases, intervention and reduction of the burden of other non-communicable neurological disorders is much more complex and requires the development of new evidence to design more effective treatments and prevention measures. Further epidemiological studies on neurological disorders in various populations are required to fill the gaps in the knowledge of distribution, frequency, outcomes, and determinants of major neurological disorders. We call on a wide range of clinicians, surgeons, and other health-care professionals with neurological expertise caring for individuals with neurological disorders to work collaboratively with the GBD to strengthen the accuracy of future GBD estimates by enhancing epidemiological research of neurological disorders.
