# Supplementary material for: Global, regional, and national burden of neurological disorders, 1990–2016: a systematic analysis for the Global Burden of Disease Study 2016
Source: Lancet Neurol. 2019 May;18(5):459–80. doi: 10.1016/S1474-4422(18)30499-X (PMC6459001; doi:10.1016/S1474-4422(18)30499-X)
Supplement: Supplementary appendix [file mmc1.pdf]

THE LANCET
Neurology

Supplementary appendix

This appendix formed part of the original submission and has been peer reviewed. We post it as supplied by the authors.

Supplement to: GBD 2016 Neurology Collaborators. Global, regional, and national burden of neurological disorders, 1990–2016: a systematic analysis for the Global Burden of Disease Study 2016. *Lancet Neurol* 2019; published online March 14. http://dx.doi.org/10.1016/S1474-4422(18)30499-X.

SUPPLEMENTARY APPENDIX

Figure. GBD regions and super-regions

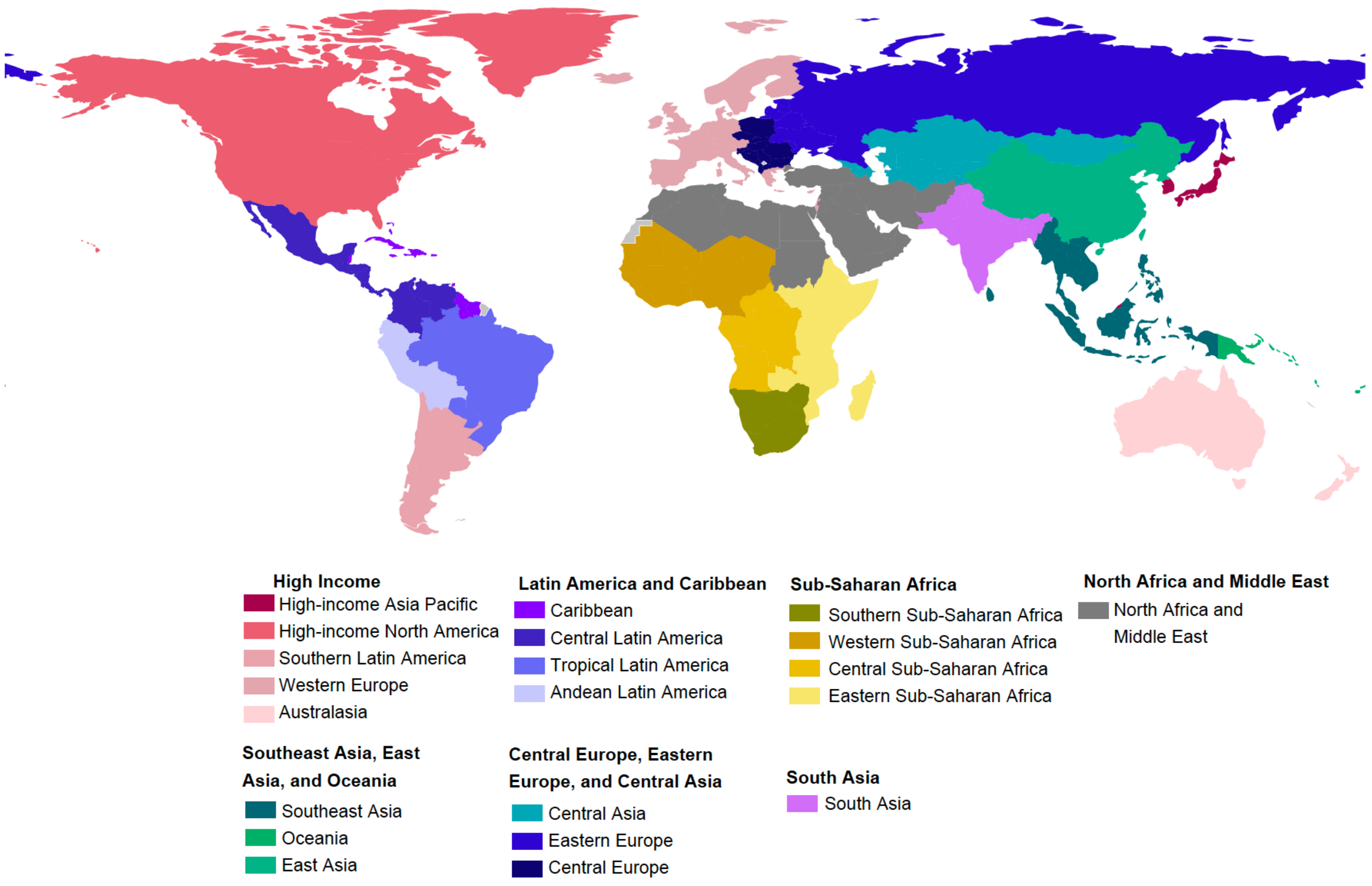

DETAILS ON THE METHODS OF ESTIMATES FOR TETANUS, ENCEPHALITIS AND THE RESIDUAL CATEGORY OF OTHER NEUROLOGICAL DISORDERS

Tetanus

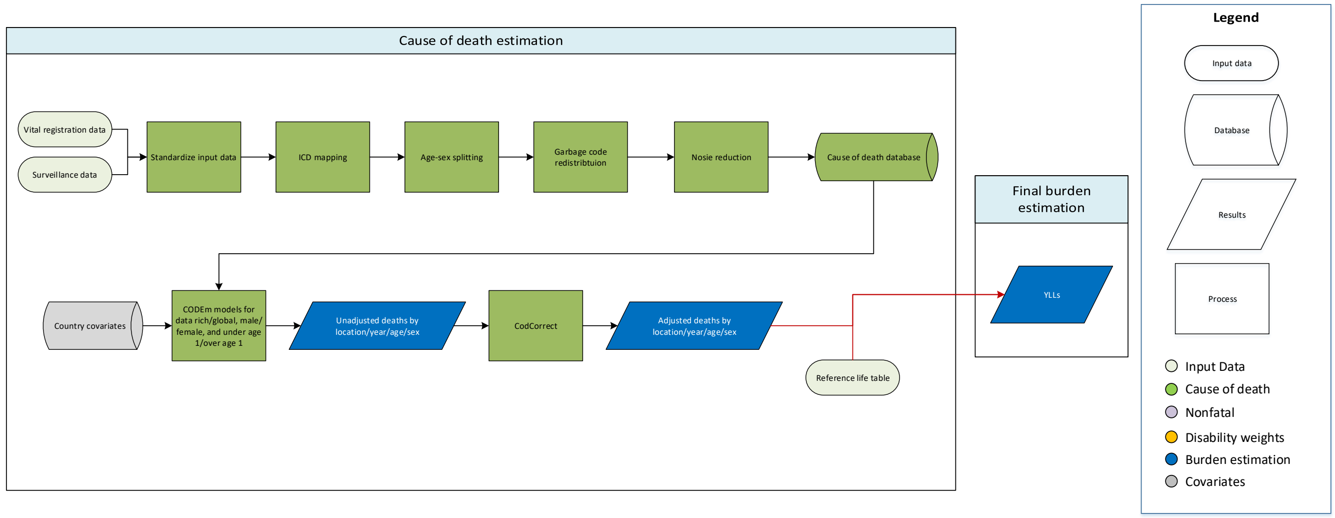

Input data

Mortality data from vital registration, verbal autopsy, and surveillance sources were used. Data were outliered if they largely conflicted with the majority of data from other studies conducted either in the same or different countries with similar sociodemographic characteristics in the same region.

Modeling strategy

A general CODEm modeling strategy was used. We ran separate models for under 1 year and 1 to 95+ years. There were no substantive changes in modeling strategy from GBD 2015.

Flowchart

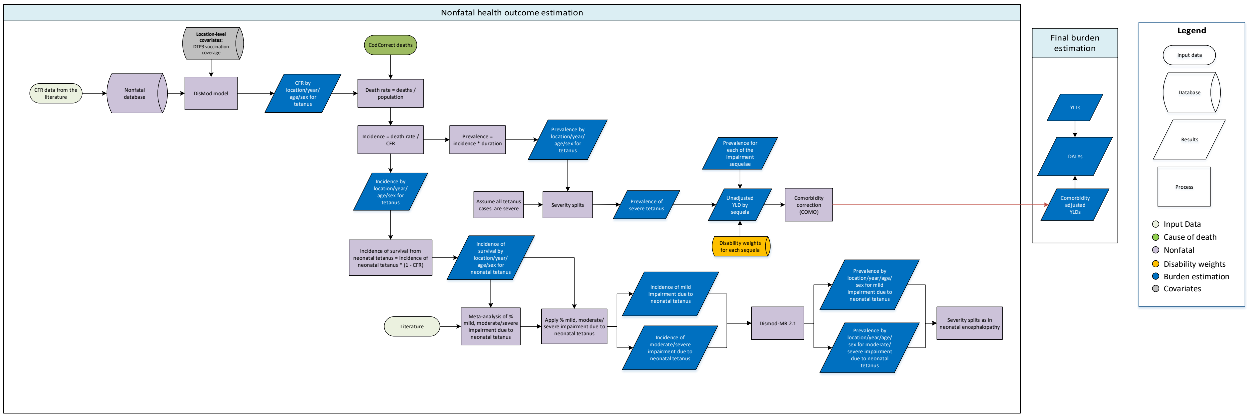

Case definition

Tetanus is a serious bacterial disease caused by the bacterium *Clostridium tetani.* For tetanus, the ICD 10 codes are A33-A35.0, Z23.5, and ICD 9 codes are 037-037.9, 771.3, V03.7.

Input data

Model inputs

For GBD 2016, input data for the estimation of tetanus included case fatality rate data extracted from a systematic review the literature and IHME tetanus mortality estimates calculated with CODEm.

A systematic review was conducted for GBD 2016. The PubMed search terms were: (tetanus[Title/Abstract]) AND (case fatality[Title/Abstract]) AND ("2013"[Date - Publication]: "2016"[Date - Publication]).

Severity split & disability weights

We assume that all tetanus cases are severe episodes of acute infectious diseases. The lay descriptions and disability weights for tetanus derived from the GBD Disability Weights study are shown below.

Table 1. Severity splits, lay descriptions, and disability weights (DW)

Severity level	Lay description	DW (95% CI)
Severe	Has a high fever and pain, and feels very weak, which causes great difficulty with daily activities.	0.133 (0.088–0.19)

Regarding the severity level of impairment due to neonatal tetanus, we assume the same distribution as in neonatal encephalopathy.

Modelling strategy

We used DisMod-MR 2.0 as a meta-regression tool to pool the case fatality data and generate location-year-age-sex-specific case fatality rate estimates. We used DTP3 coverage as a location-level covariate. Mortality was modelled using the standard CODEm tool on neonatal tetanus (ages 0-0.1) and non-neonatal tetanus (ages 1-80) separately for males and females. Incidence was then calculated as:

$$\text{Incidence} = \text{mortality rate} / \text{case fatality rate}$$

Prevalence was then computed based on the estimated incidence and duration draws derived from literature review.

To estimate mild and moderate impairment due to neonatal tetanus, we first computed the incidence of survival from neonatal tetanus as:

$$\text{Incidence of survival} = \text{incidence} * (1 - \text{CFR})$$

We then conducted a meta-analysis of published studies to estimate the proportion of mild impairment due to neonatal tetanus and moderate-to-severe impairment due to neonatal tetanus. We applied these proportions to the estimated incidence of survival, to generate incidence of mild impairment due to neonatal tetanus and moderate-to-severe impairment due to neonatal tetanus, which were used as input

data in DisMod 2.0. We ran two separate DisMod models (one for mild impairment due to neonatal tetanus, and one for moderate-to-severe impairment due to neonatal tetanus) to generate age-sex-year-country-specific estimates.

The table below shows betas and exponentiated values for the covariates used in the estimation process (from the DisMod case-fatality model), which can be interpreted as an odds ratio.

Table 3. Beta and exponentiated beta values

Covariate	Parameter	Beta (95% CI)	Exponentiated beta (95% CI)
DTP3 coverage (proportion)	Case fatality	0.52 (-0.061 — 1.32)	1.68 (0.94 — 3.76)
Sex	Case fatality	-0.12 (-0.35 — 0.10)	0.89 (0.71 — 1.11)

No other significant changes were made to the modelling strategy for GBD 2016.

Encephalitis

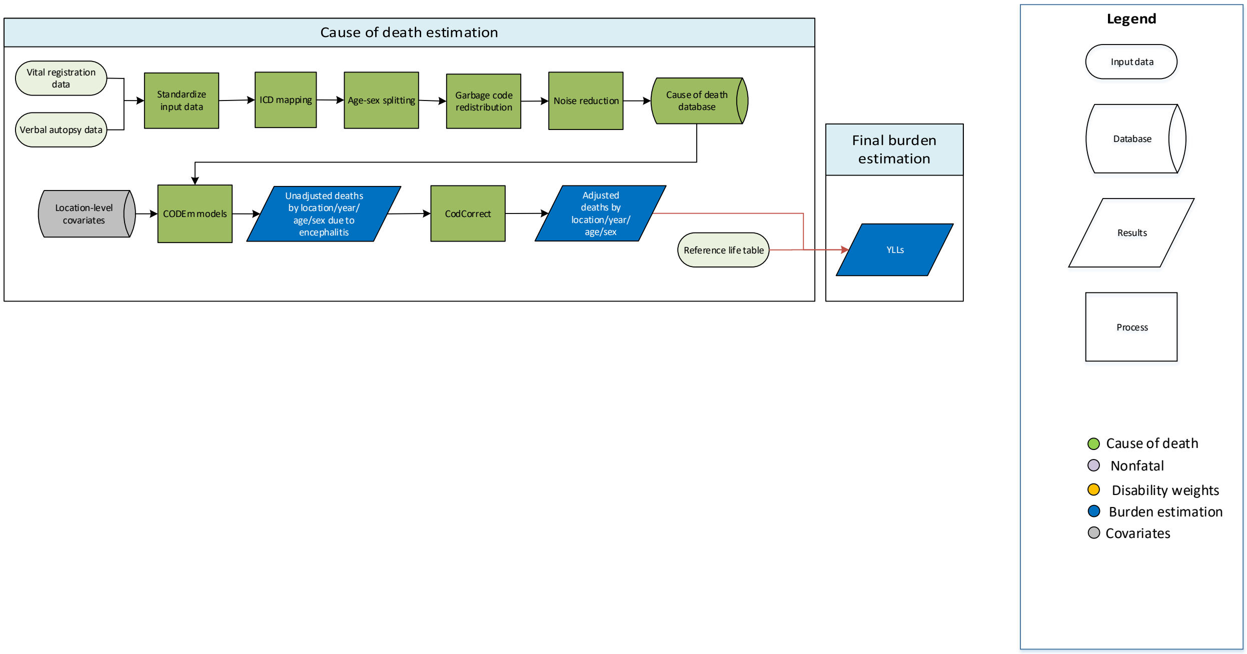

Input data

For GBD 2016, vital registration (VR) and verbal autopsy (VA) data were used to model this cause. We outliered data in instances where garbage code redistribution and noise reduction, in combination with small sample sizes, resulted in unreasonable cause fractions when compared to regional, super-regional, and global rates, and data that violated well-established time or age trends. Outliering methods were consistent across both vital registration and verbal autopsy data.

Modelling strategy

We modelled deaths due to encephalitis with a standard CODEm model using the cause of death database and location-level covariates as inputs. We hybridized separate global and data-rich models to acquire unadjusted results, which were adjusted using CodCorrect to reach final years of life lost (YLLs) due to encephalitis.

In GBD 2013, the encephalitis model was modelled using two age categories – under-5 and 5 years and above – because the mortality trends differed substantially between children and adults and a significant number of data sources only had data for under-5-year-olds. With the addition of new data sources for GBD 2015, this modelling process was deemed unnecessary and the encephalitis model covered the entire age range. Another significant change was the addition of the Japanese encephalitis covariate, which is a binary covariate indicating if the location is known to be endemic for Japanese encephalitis. The covariate was modelled according to data from the Centers for Disease Control and Prevention.[1]

We made no other significant changes to input data or modelling strategy for GBD 2016.

Flowchart

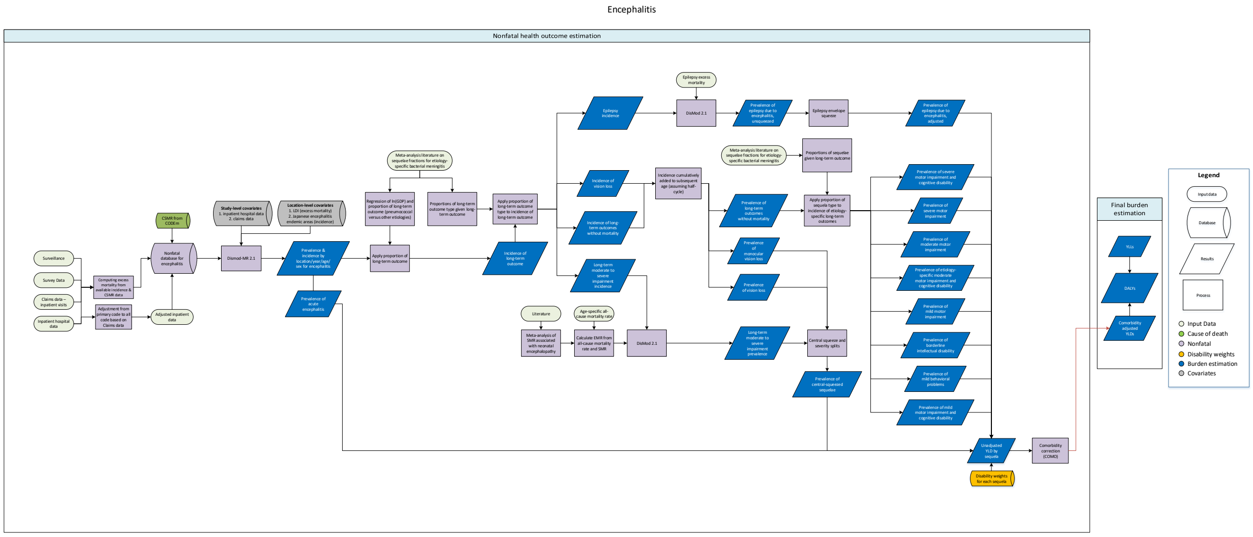

Case definition

Encephalitis is a disease caused by an acute inflammation of the brain. Symptoms of encephalitis can include flu-like symptoms like headache, fever, drowsiness, and fatigue, and at times, seizures, hallucinations, or stroke. Included in the GBD modeling were cases meeting ICD-10 diagnostic criteria for encephalitis (A83-A86.4, B94.1, F07.1, G04-G05.8) (1).

Input data

Model inputs

In the GBD 2010 study, a systematic review of literature was conducted to capture studies of incidence, excess mortality rate, remission, and standardized mortality ratio for encephalitis. These data sources included hospital data and literature. The inclusion criteria stipulated that: (1) the publication year must be between 1980 and 2010; (2) sufficient information must be provided on study method and sample characteristics to assess the quality of the study; and (3) study samples must be representative of the general population. No limitation was set on the language of publication. For GBD 2013, the GBD 2010 search strategy was replicated to capture epidemiological studies published between 2010 and 2013. We did not do a literature review for GBD 2016.

Additional sources we included in the acute bacterial meningitis model were inpatient-only hospital data and US claims data from 2000, 2010, and 2012, primary diagnosis and inpatient only. Sequelae and severity splits were informed by a meta-analysis, Edmond et al (2), while an internal meta-analysis informed mortality estimates for long-term moderate-to-severe impairments (3).

Data were outliered or excluded if we found they differed significantly when compared to regional, super-regional, and global rates.

The tables below show the number studies included in GBD 2016, as well as the number of countries or subnational units and GBD world regions represented for the encephalitis.

Table 1. Acute encephalitis

	Prevalence	Incidence
Studies	0	26
Countries/subnationals	0	37/291
GBD world regions	0	16

Modeling strategy

Non-fatal outcomes were modeled using a combination of custom models and DisMod-MR 2.2, with minor changes from the GBD 2015 modeling process. First, the overall incidence and prevalence of encephalitis was modeled to estimate the short-term morbidity due to acute infection. This DisMod model had a set duration (1/remission) between 2.9 and 3.1 weeks. We also imposed caps on excess mortality for ages 10-50, and a cap on incidence from ages 10-100. US claims data and literature data were flagged with year-specific covariates to be crosswalked to the reference data, which were inpatient-only, primary diagnosis hospital data. We used the function in DisMod-MR 2.1 to pull in cause-specific mortality rate (CSMR) data from our CODEm and CODcorrect analyses and match with incidence data points for the same geography. We calculated excess mortality rate to estimate priors by dividing CSMR by prevalence, calculated from remission and incidence. To help inform trends where we lack data, we applied a binary country-level covariate at the subnational and country level that indicates if the location is in a Japanese Encephalitis endemic area (4). We forced a positive relationship, with a lower bound of 0 and an upper bound of 0.1. We also applied a lag-distributed income covariate to excess mortality, log transformed and forced negative with an upper bound of 0 and a lower bound of -1. Betas and exponentiated values (which can be interpreted as an odds ratio) are shown in the tables below for study-level covariates and country-level covariates.

Table 2. Study covariates

Study covariate	Parameter	beta	Exponentiated beta
Claims data – 2000	Incidence	-0.81 (-0.84 to – 0.78)	0.44 (0.43 – 0.46)
Claims data – 2010	Incidence	-0.56 (-0.59 to – 0.54)	0.57 (0.55 – 0.58)
Claims data – 2012	Incidence	-0.45 (-0.47 to -0.42)	0.64 (0.62 – 0.65)
Literature	Incidence	-1.23 (-1.36 to -1.13)	0.29 (0.26 – 0.32)

Table 3. Country-level covariates

Country-level covariate	Parameter	beta	Exponentiated beta
Japanese Encephalitis endemic area (binary)	Incidence	0.068 (0.030 – 0.097)	1.07 (1.03 – 1.10)
LDI (log transformed)	Excess mortality	-0.015 (-0.055 to -0.0002)	0.99 (0.95 – 1.00)

In addition to short-term sequelae as a result of acute encephalitis, we also modeled the long-term outcomes from encephalitis.

Sequelae Splits

We first split the long-term sequelae among survivors of acute infection. We calculated the acute phase survivors by applying the excess mortality (calculated by the acute meningitis DisMod model) to the incidence of each etiology (excess mortality was converted to case fatality rate by $e^{(\text{-excess mortality x 1/(excess mortality + remission))}}$). The survivors were then subject to long-term sequelae by applying the post-discharge proportions of health consequences calculated by a meta-analysis by Edmond et al (2). We calculated the ratio of acute encephalitis survivors that result in a major long-term impairment, and the ratio of minor impairments to major impairments, based off a regression of log-transformed GDP and ratio values from Edmonds et al. The regression is shown below:

$$y = -0.33590 \ln(GDP) + 1.15230$$

We assumed a similar pattern of health outcomes for encephalitis infection survivors as with other bacterial meningitis survivors (except hearing loss, as we could not find evidence of hearing loss as a consequence of encephalitis infection). We used these two ratios to calculate the proportions of survivors who contract a long-term minor impairment and those who contract a long-term major impairment. The proportion with major impairments were further split (again using pooled proportions from Edmond et al) into specific major impairments, which were grouped into vision loss, moderate to severe cognitive impairments, and epilepsy.

The calculated incidence of long-term sequelae was then converted to prevalence by two different approaches. For the sequelae not associated with excess mortality, which were vision loss, intellectual disability, motor impairment, and behavioral problems, the incidence of each age was cumulatively added up to the subsequent age (assuming half-cycle) to construct prevalence at each age. If the sequela is associated with excess mortality (epilepsy and moderate-to-severe cognitive impairments), the calculated incidence was uploaded into DisMod together with the corresponding mortality parameters (excess mortality data from the epilepsy envelope DisMod model, and standardized mortality ratio data from a neonatal encephalopathy meta-analysis, converted to excess mortality using all-cause mortality estimates) to estimate the prevalence. Vision loss and epilepsy estimates were squeezed and severity split centrally.

Disability weights

The basis of the GBD disability weight survey assessments is lay descriptions of sequelae highlighting major functional consequences and symptoms. The lay descriptions and disability weights for sequelae associated with encephalitis are shown below.

Table 4. Severity splits, lay descriptions, and DWs

Severity split	Lay description	DW (95% CI)
Mild behavior problems	This person is hyperactive and has difficulty concentrating, remembering things, and completing tasks.	0.045 (0.028-0.066)

Moderate motor impairment	This person has some difficulty in moving around, and difficulty in lifting and holding objects, dressing and sitting upright, but is able to walk without help.	0.061 (0.04-0.089)
Moderate motor plus cognitive impairments	This person has some difficulty in moving around, holding objects, dressing and sitting upright, but can walk without help. This person has low intelligence and is slow in learning to speak and to do simple tasks.	0.203 (0.134-0.29)
Long- term mild motor impairment	This person has some difficulty in moving around but is able to walk without help.	0.01 (0.005-0.02)
Borderline intellectual disability	This person is slow in learning at school. As an adult, the person has some difficulty doing complex or unfamiliar tasks but otherwise functions independently.	0.011 (0.005-0.02)
Severe motor impairment	This person is unable to move around without help, and is not able to lift or hold objects, get dressed, or sit upright.	0.402 (0.268-0.545)
Epilepsy	(combined DW)	NA
Blindness	Is completely blind, which causes great difficulty in some daily activities, worry and anxiety, and great difficulty going outside the home without assistance.	0.187 (0.124-0.26)
Acute encephalitis	This person has a high fever and pain, and feels very weak, which causes great difficulty with daily activities.	0.133 (0.088-0.19)
Mild intellectual disability	This person has low intelligence and is slow in learning at school. As an adult, the person can live independently but often needs help to raise children and can only work at simple supervised jobs.	0.043 (0.026-0.065)
Monocular distance vision loss	This person is blind in one eye and has difficulty judging distances	0.017 (0.009-0.029)
Mild motor plus cognitive impairments	This person has some difficulty in moving around but is able to walk without help. The person is slow in learning at school. As an adult, the person has some difficulty doing complex or unfamiliar tasks but otherwise functions independently.	0.031 (0.018-0.05)
Severe motor plus cognitive impairments	This person cannot move around without help, and cannot lift or hold objects, get dressed or sit upright. The person also has very low intelligence, speaks few words, and needs constant supervision and help with all daily activities.	0.542 (0.37-0.702)

No other significant changes were made to the modeling process for GBD 2016.

Other Neurological Disorders

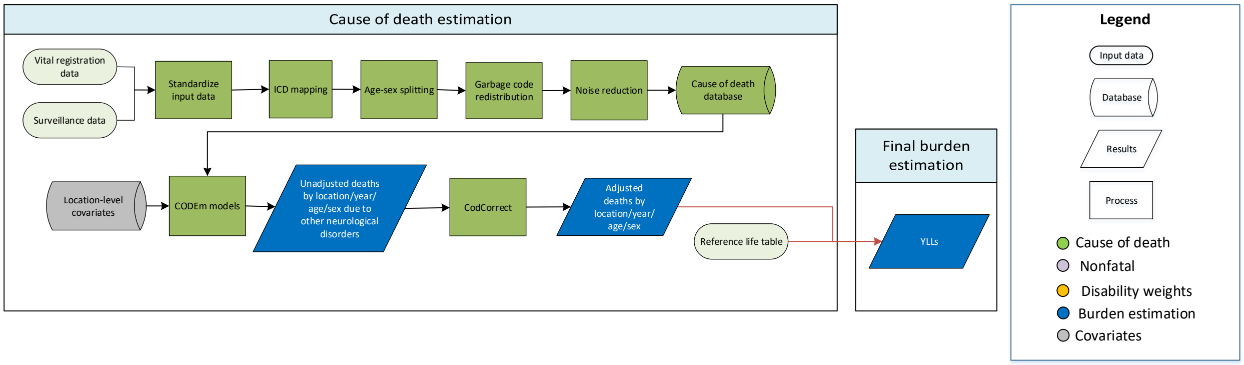

Input data

Data used to estimate other neurological disorders included vital registration and surveillance data from the cause of death (COD) database. Our outlier criteria were to exclude data points that (1) were implausibly high or low, (2) substantially conflicted with established age or temporal patterns, or (3) significantly conflicted with other data sources conducted from the same locations or locations with similar characteristics (ie, Socio-demographic Index).

Modelling strategy

The standard CODEm modelling approach was applied to estimate deaths due to other neurological disorders. Male and female CODEm models were run for deaths occurring between ages 28 days to 95+ years. For GBD 2016, the fruit and meat intake per capita covariates were adjusted to reflect intake per 2,000 kcal per day diet. Additionally, the covariate health system access was replaced with the health care access and quality index. There were no other significant changes from the GBD 2015 modelling strategy. The covariates used are displayed below.

**Level**	**Covariate**	**Direction**
1	underweight proportion was under 2 standard deviations	+
	mean body mass index	+
	mean cholesterol	+
	mean systolic blood pressure	+
	pig meat consumption (kcal per capita)	+
2	alcohol (litres per capita)	+
	animal fat consumption (kcal per capita)	+
	health care access and quality index	-
	fruit consumption adjusted	-
	population density over 1,000 per square kilometer pct	+
	red meat consumption adjusted	+
3	cumulative cigarette consumption (10 years)	+
	cumulative cigarette consumption (5 years)	+
	education (years per captia)	-
	log-transformed LDI (per capita)	-
	smoking prevalence	+

	Socio-demographic Index	0

In addition to the neurological disorders described above, there are many diverse types of neurological disorders with a range of severities and associated sequelae. Because these neurological disorders are diverse in their underlying causes and risk factors as well as in their associated health outcomes, modelling them together in a DisMod-MR model would not produce reliable estimates of prevalence or excess mortality. Instead, we calculated the YLDs caused by neurological disorders directly using a YLD/YLL ratio.

We calculated the ratio of YLDs to YLLs for Alzheimer's disease and other dementias, Parkinson's disease, idiopathic epilepsy, multiple sclerosis, and motor neuron diseases. We then multiplied this YLD/YLL ratio by the YLL estimates for other neurological disorders from the GBD 2016 CoD analysis, providing us with a placeholder estimate of the YLDs associated with other neurological disorders.

These methods write-ups have previously been published in appendices of:

- Naghavi M, Abajobir AA, Abbafati C, et al. Global, regional, and national age-sex specific mortality for 264 causes of death, 1980-2016: a systematic analysis for the Global Burden of Disease Study 2016. *The Lancet* 2017; **390**(10100): 1151-210.
- Vos T, Abajobir AA, Abate KH, et al. Global, regional, and national incidence, prevalence, and years lived with disability for 328 diseases and injuries for 195 countries, 1990-2016: a systematic analysis for the Global Burden of Disease Study 2016. *The Lancet* 2017; **390**(10100): 1211-59.
